# Opposing cortical forces: Alpha slowing and sensorimotor mu acceleration during motor-related BCI training

**DOI:** 10.1371/journal.pcbi.1014112

**Published:** 2026-04-01

**Authors:** Kyriaki Kostoglou, Gernot R. Müller-Putz

**Affiliations:** 1 Institute of Neural Engineering, Graz University of Technology, Graz, Austria; 2 BioTechMed, Graz, Austria; University of Dundee, UNITED KINGDOM OF GREAT BRITAIN AND NORTHERN IRELAND

## Abstract

Brain-computer interfaces (BCIs) depend on the reliable decoding of brain activity, yet key rhythms like alpha and mu are not spectrally static and can shift with cognitive and motor demands. Here, we investigated within-session changes in instantaneous alpha/mu frequency and magnitude during motor-related BCI calibration using an oscillator-tracking framework based on an extended Kalman filter (EKF). We applied this method to four public EEG datasets spanning motor execution and imagery tasks. Across all datasets, we observed consistent increases in mu instantaneous frequency and magnitude over central sensorimotor regions, indicative of motor engagement and possible training-related neuroplasticity. In contrast, posterior and surrounding cortical areas often showed alpha slowing, suggestive of declining vigilance or cognitive fatigue, or alternatively, resource reallocation via inhibition of task-irrelevant regions. These opposing spatial trends underscore the functional heterogeneity of alpha-band activity across the cortex. Our results highlight the potential of real-time frequency tracking not only to improve decoding accuracy but also to monitor neurophysiological state changes and guide adaptive adjustments in BCI calibration paradigms.

## Author summary

Brain-computer interfaces (BCIs) convert brain activity into commands for external devices, with applications ranging from motor rehabilitation to assistive communication. A key challenge is that the brain’s rhythmic activity can shift in frequency and strength during use. In particular, alpha rhythms (around 8–12 Hz) dominate posterior brain regions and are linked to attention and visual processing, while mu rhythms, a variant of alpha over the sensorimotor cortex, are closely tied to movement preparation and execution. Changes in these rhythms can directly affect BCI accuracy, yet until now, no study has tracked them continuously during training. We developed a mathematical tracking method to follow moment-to-moment changes in the frequency and magnitude of alpha and mu rhythms. Applying it to four EEG datasets, we uncovered a striking, previously unreported dissociation: mu oscillations tended to speed up over the motor cortex, while alpha slowed down in non-motor cortical areas. This pattern likely reflects distinct neural processes, such as motor learning, attention shifts, and fatigue, that evolve together during BCI use. Recognizing and adapting to these dynamics could make BCIs more robust, personalized, and effective. Our method also offers a powerful tool for studying brain rhythm adaptation in learning, cognitive effort, and clinical contexts.

## Introduction

Electroencephalography (EEG)-based brain-computer interfaces (BCIs) provide a non-invasive means of controlling external devices by decoding brain activity during movement execution (ME), motor imagery (MI), or attempted movement (MA). A key neural signature used in such systems is alpha-band activity (~8–12 Hz). Within this range, mu rhythms, a sensorimotor-specific subset of alpha, are particularly relevant for BCIs. Mu is often divided into lower mu (8–10 Hz) and upper mu (10–13 Hz) [1–3], and is known to desynchronize during motor-related processes, a phenomenon termed event-related desynchronization (ERD) [4]. As such, the modulation of mu rhythms has become a fundamental component of motor-based BCIs for both healthy users and clinical populations [5–7]. In contrast, non-sensorimotor alpha rhythms show distinct regional specializations: posterior alpha, strongest over occipital and parietal regions, is commonly linked to visual processing, attentional demands, arousal levels, and mental fatigue [8–12], while frontal alpha has been associated with executive control, mental fatigue, cognitive effort, task engagement and affective processes [3,8,13]. While mu and alpha occupy a similar frequency range, they differ in both topography and functional significance.

While most BCI research to date has focused on amplitude dynamics, such as the strength and lateralization of ERD, relatively little attention has been given to the frequency characteristics of these rhythms. Yet it is increasingly recognized that alpha/mu rhythms are not spectrally static. Instead, their peak frequencies can shift over time, reflecting both endogenous brain state changes (e.g., fatigue, cognitive engagement) and longer-term neuroplastic adaptations [11,14–16]. Alpha slowing has been linked to effort, fatigue, aging, and neurological/cognitive impairment [16–19], while faster alpha is associated with better memory [20], attention [3], and motor function [21]. Mu frequency shifts similarly relate to motor learning, efficiency, and skill [22–25]. For example, athletes and dancers show higher mu frequencies than non-experts, suggesting sensorimotor tuning [26]. Neurofeedback studies further demonstrate that people can modulate their mu peak frequency with visual or haptic feedback training, leading to modest (0.2–0.5 Hz) increases and improved motor/cognitive performance [27]. Mu frequency also serves as a marker of motor recovery: it is reduced near lesions during motor imagery in stroke patients [28], and people with spinal cord injury often show lower mu and higher beta ERD frequencies during attempted movement, especially when muscular control is absent [29]. These shifts may result from compensatory neural adaptations or a loss of sensory feedback in chronically impaired motor systems.

These findings suggest that frequency shifts may reflect adaptive changes in brain activity during BCI use. However, it remains unclear whether these shifts consistently occur during training and how they vary across regions and over time. Hülsdünker et al. [21] observed that mu frequency increased under high demands during balance tasks, indicating rhythm acceleration with sensorimotor load. Other studies reported alpha slowing over time in a visuomotor task, suggesting reduced vigilance or cortical disengagement over time [30,31]. New methods now allow more precise frequency tracking. For instance, Vidaurre et al. [32] showed that instantaneous and localized frequency changes contain task-relevant information beyond what traditional methods capture. These insights are crucial, as most BCI decoders rely on fixed frequency bands, potentially missing dynamic changes related to learning, fatigue, or engagement.

In earlier work, using cascade-based time-varying autoregressive (TV-AR) pole tracking [33], we found that mu ERD during motor tasks often co-occurred with frequency increases, suggesting that magnitude suppression and frequency acceleration jointly reflect motor engagement. This led us to examine how posterior alpha and mu rhythms evolve during BCI calibration, hypothesizing that motor regions would show increasing frequency and magnitude from neuroplastic adaptation, while posterior and surrounding areas might exhibit alpha slowing arising from fatigue-related state changes and/or functional inhibition of task-irrelevant areas. To investigate this, we developed an enhanced oscillator-tracking method modeling EEG rhythms as outputs of time-varying second-order TV-AR systems. Our previous cascade-form TV-AR method [33,34] could track broadband content but lacked rhythm selectivity. In contrast, the new model uses an extended Kalman Filter (EKF) to track instantaneous frequency and damping directly as latent variables, allowing stable, narrowband, and physiologically interpretable tracking of alpha and mu rhythms.

## Results

**Fig 1:**
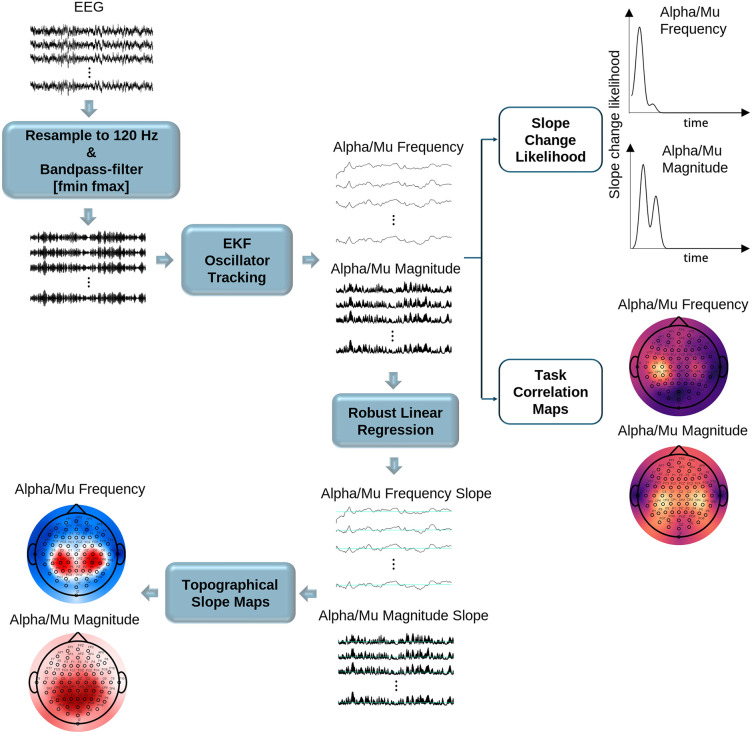
Overview of the EEG analysis pipeline. After preprocessing, including ICA artifact removal, the cleaned EEG data were resampled and bandpass filtered to isolate the alpha/mu range. EEG data was then processed using an EKF-based oscillator tracking algorithm to estimate instantaneous frequency and magnitude for each channel. Robust linear regression was then applied to compute the slope of each feature across time, yielding topographical maps of alpha/mu frequency and magnitude changes (topographical slope maps). To ensure that these linear slopes captured sustained trends rather than transient fluctuations, a slope change likelihood curve was derived for both frequency and magnitude. The optimal filter configuration (cutoff frequencies and filter order) was selected by computing the correlation between EKF-estimated mu-band magnitude and task-related reference signals (e.g., binary motor labels or continuous kinematic traces), and identifying the band that maximized average absolute correlation across channels. These correlations were also visualized as topographical maps (task correlation maps), highlighting the spatial distribution of task-relevant modulation and aiding interpretation of the functional relevance of alpha/mu dynamics.

Following EEG preprocessing (see Materials and methods), we applied the extended Kalman Filter (EKF) tracking pipeline (Fig 1) to all datasets listed in Table 1 (see Materials and methods). EEG signals were bandpass filtered using multiple alpha-range configurations, and the band yielding the highest absolute correlation between EKF-estimated mu magnitude and task reference signals (e.g., task vs. rest, movement labels, kinematics) over all participants was selected to best capture mu dynamics (i.e., [8 12] Hz for Schalk2004, [8 13] Hz for Dreyer2023, [8 11] Hz for Schwarz2020 and [8 12] Hz for Pulferer2022).

**Table 1:** EEG dataset description. All EEG recordings were obtained from non-disabled participants.

Dataset	Reference	Description	# EEG Channels	Sampling Rate (Hz)	# Participants	Duration	Motor Task
Schalk2004	[54] https://doi.org/10.13026/C28G6P	execution and imagination of unilateral and bilateral hand and foot movements, including opening and closing fists and feet	64	160	109	~25 min session including both ME and MI	ME and MI
Dreyer2023	[55] https://doi.org/10.5281/zenodo.8089820	left- and right-hand motor imagery using the Graz BCI protocol	22	512	87	Two successive ~7-min MI sessions (acquisition phase)	MI
Schwarz2020	[56] https://bnci-horizon-2020.eu/database/data-sets #27	execution of natural reach‑and‑grasp actions	58	256	15	~20 min ME session	ME
Pulferer2022	[57] https://bnci-horizon-2020.eu/database/data-sets #32	performing a target-tracking/shape-tracing task on-screen	60	200	10	Three ~50 min sessions within 5 days	Suppressed ME (due to physical restraint)

**Fig 2:**
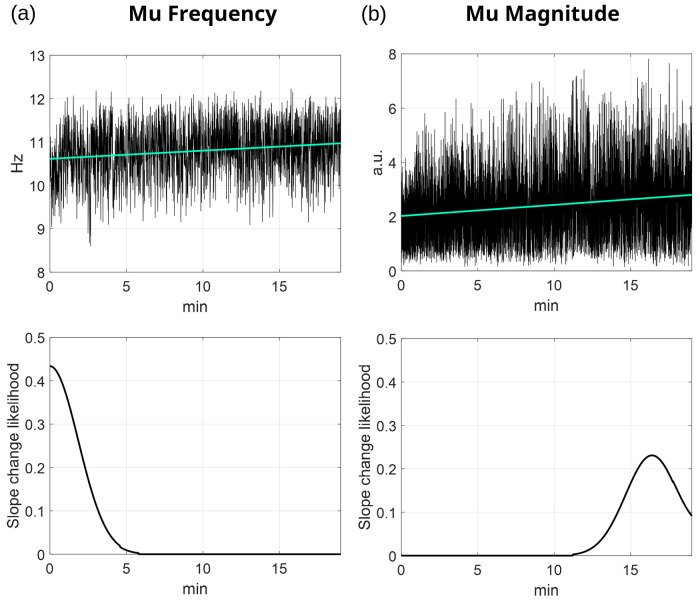
Representative example of EKF-tracked mu (a) frequency and (b) magnitude from channel C3 of a participant in the Schalk2004 dataset. The top panels display the raw estimated trajectories (black) along with the best-fit linear trend (cyan). The estimated slope of the mu frequency was 1.128 Hz/hour (*p* < 0.05, after correction for multiple comparisons), while the mu magnitude increased at a rate of 2.458 a.u./hour (*p* < 0.05, after correction for multiple comparisons). The bottom panels show the slope change likelihood curves, quantifying the probability of abrupt trend shifts over time based on binary change point detection smoothed with a noncausal Gaussian kernel. In the mu frequency panel (bottom left), the slope change likelihood sharply peaks near the session start (~0–2 minutes), indicating a prominent early change in slope. This is consistent with the visible shift in the frequency trajectory shown in the top-left panel. In contrast, the mu magnitude panel (bottom right) exhibits a delayed and more gradual peak in slope change likelihood around 14–16 minutes. Visually inspecting (b) suggests that mu magnitude started stabilizing toward the session’s end.

Fig 2 illustrates the evolution of mu frequency and magnitude in a representative participant from the Schalk2004 dataset (channel C3). EKF-derived trajectories show frequency and magnitude increasing by 1.13 Hz/hour and 2.46 arbitrary units/hour (a.u./hour), respectively. To validate the assumption that these changes were sustained (i.e., monotonic), we applied a slope change detection algorithm (see Materials and Methods) identifying the most prominent inflection point per time series. The resulting slope change likelihood curves (bottom panels) show the probability of such inflection points across the session. In this example, likelihood peaks appeared near the session boundaries, suggesting that the main trends were not interrupted by mid-session reversals. Practically, for each participant, we visualized the slope change likelihood averaged across all channels. This helped verify that no substantial intermediate changes occurred during the session, reinforcing the reliability and unbiased nature of the reported slope estimates.

**Fig 3:**
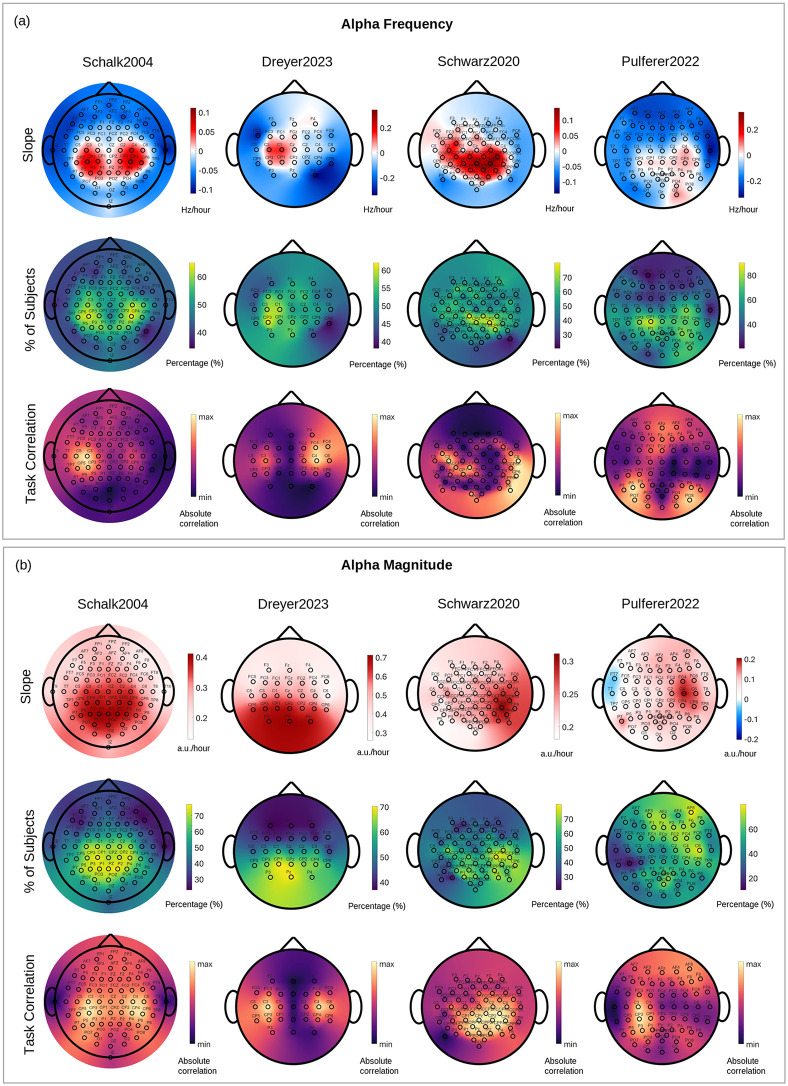
(a) Grand-average EEG topographical maps of alpha/mu-band frequency slopes (top row), the percentage of subjects exhibiting a positive slope at each channel (middle row), and task correlations (bottom row), and (b) the corresponding maps for alpha/mu-band magnitude for the Schalk2004, Dreyer2023, Schwarz2020, and Pulferer2022 datasets (left to right; columnwise). In the slope maps (top rows in (a) and (b)), red hues indicate increasing trends over time (i.e., rising frequency in Hz/hour or magnitude in a.u./hour), while blue hues indicate decreasing trends. The percentage maps (middle rows in (a) and (b)) depict, for each electrode, the fraction of participants exhibiting a statistically significant positive slope (i.e., increasing frequency or magnitude over time), providing a channel-wise measure of how consistently the direction of change is observed across individuals. The task correlation maps (bottom rows in (a) and (b)) show the spatial distribution of the absolute correlation between EKF-estimated alpha/mu-band dynamics and task-related reference signals (e.g., task vs. rest, movement labels, kinematics), averaged across participants. This correlation analysis was used to identify the frequency range most strongly modulated by the task, representing the functionally relevant mu rhythm. Topoplots were generated using the MATLAB toolbox from Víctor Martínez-Cagigal (2025): Topographic EEG/MEG plot (https://www.mathworks.com/matlabcentral/fileexchange/72729-topographic-eeg-meg-plot).

Fig 3 summarizes the main EKF tracking results. Fig 3a presents grand-average topographies of alpha/mu frequency slopes (top, in Hz/hour), the percentage of subjects exhibiting a positive frequency slope (middle), and correlations between alpha/mu frequency and task events (bottom). Fig 3b shows the corresponding maps for alpha/mu magnitude (in a.u./hour), including slope, percentage of positive slopes, and task-related correlations. In the slope maps, red hues indicate increasing trends over time, whereas blue hues indicate decreasing trends. Across all datasets, mu frequency increased focally over central regions, whereas non-sensorimotor alpha slowed. Magnitude increased more broadly but remained strongest over central areas.

The percentage maps (middle rows in Figs 3a,b) complement these findings by quantifying, for each electrode, the proportion of participants exhibiting a statistically significant positive slope, thereby capturing the spatial consistency of the direction of change across individuals. For frequency (Fig 3a), these maps indicate that a large proportion of participants, often exceeding 60–80% of subjects at central sensorimotor electrodes, exhibited positive slopes, demonstrating a highly consistent increase in mu frequency across individuals. For magnitude (Fig 3b), positive slopes were present across a broader spatial extent, including both central and posterior regions, with a high proportion of participants, again frequently exceeding 60–80%, exhibiting increases in alpha/mu magnitude over the course of the session.

The task-correlation maps (bottom rows in Figs 3a,b) illustrate the spatial distribution of the absolute correlation between EKF-estimated alpha/mu-band dynamics and task-related reference signals, averaged across participants. These correlations were used to guide the selection of dataset-specific alpha/mu frequency bands for subsequent analyses. Across all datasets, the strongest task-related absolute correlations were localized over sensorimotor regions, confirming the behavioral relevance of the observed frequency and magnitude dynamics.

**Fig 4:**
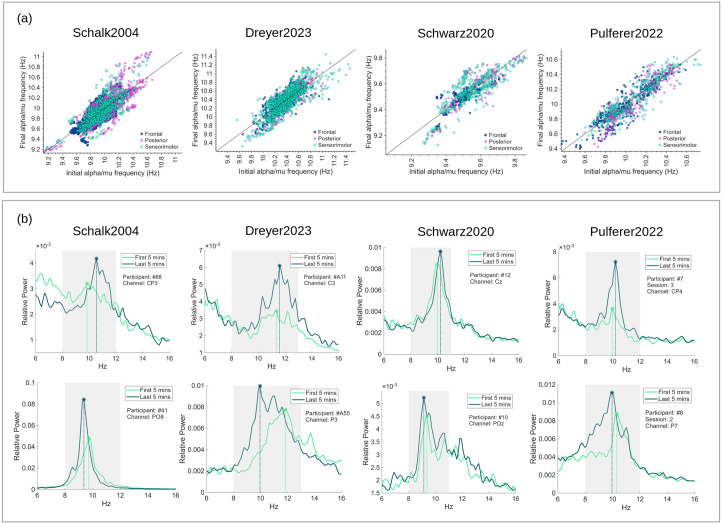
(a) Scatter plots of estimated initial versus final alpha/mu frequency for all participants across the four datasets (Schalk2004, Dreyer2023, Schwarz2020, and Pulferer2022) and (b) PSDs from representative participants and channels, comparing the first and last 5 minutes of the BCI session. In (a) initial and final frequencies were derived from the fitted robust linear regression of EKF-estimated frequency trajectories by evaluating the regression model at the beginning and at the end of each recording, rather than using instantaneous frequency estimates. For visualization, channels were grouped into anatomically defined regions of interest (ROIs: frontal, sensorimotor, and posterior; with the sensorimotor ROI comprising central and centroparietal electrodes). The identity line is shown for reference; points above the line indicate an increase in alpha/mu frequency over the session, whereas points below indicate a decrease. In (b) the top row illustrates cases where both mu peak frequency and magnitude increase over time, while the bottom row shows a decrease in mu peak frequency accompanied by a magnitude increase. Gray shaded regions indicate the dataset-specific alpha/mu frequency bands used for bandpass filtering.

To further contextualize these session-long trends, Fig 4a reports initial and final alpha/mu frequency values derived directly from the fitted session-long regression model across all participants and channels. For visualization, channels were grouped into anatomically defined regions of interest (ROIs: frontal, sensorimotor, and posterior), with the sensorimotor ROI comprising central and centroparietal electrodes. In parallel, Fig 4b shows representative power spectral densities (PSDs) from selected channels and participants, comparing the first and last 5 minutes of each BCI session. PSDs were computed using Welch’s method (5 s Hamming windows, 50% overlap) and normalized by total power in the 0.5–60 Hz range.

We next examined whether session-long slope values differed systematically across electrodes. Significant inter-electrode differences were observed only in the Schalk2004 dataset; no effects survived correction in the remaining datasets. Consequently, this analysis is reported in the Supporting Information (EEG Data – Pairwise Inter-channel Slope Comparisons, S4 Fig). As illustrated in S4 Fig, pairwise slope contrasts revealed a clear spatial dissociation between alpha/mu frequency and magnitude dynamics and enabled the identification of two sensorimotor-related regional clusters: a central and a centroparietal group. For frequency, the strongest increases were observed over centroparietal electrodes, which exhibited significantly larger positive slopes than frontal, temporal and posterior (i.e., parietooccipital and occipital) regions. Central electrodes differed mainly from frontal and temporal sites, with no significant differences relative to posterior regions. This indicates that maximal mu frequency acceleration was centered over centroparietal rather than strictly central areas, in line with the topographical patterns shown in Fig 3a. In contrast, magnitude increases were spatially more widespread: both central and centroparietal regions showed larger slopes than the frontal cortex, and centroparietal electrodes also exceeded posterior regions. Central electrodes, however, showed only limited differences relative to posterior sites, suggesting a more gradual spatial gradient for amplitude changes.

**Fig 5:**
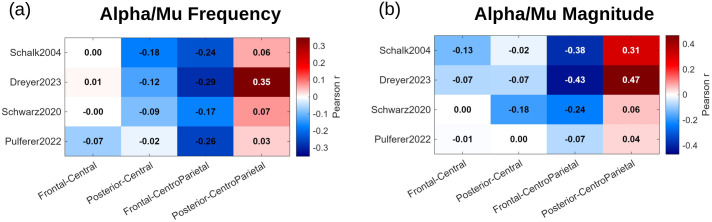
ROI-level correlation matrices summarizing inter-individual coupling of session-long alpha/mu dynamics across cortical regions for (a) alpha/mu frequency slopes and (b) alpha/mu magnitude slopes. Rows correspond to datasets, and columns indicate pairwise correlations between regional slopes (frontal–central, posterior–central, frontal–centroparietal, and posterior–centroparietal). Only correlations surviving multiple-comparison correction were retained. Significant channel-wise correlations were subsequently averaged within ROIs. Color encodes the Pearson correlation coefficient across participants, with red indicating positive coupling and blue indicating inverse coupling. The matrices reveal consistent cross-dataset patterns, including inverse relationships between sensorimotor (central/centroparietal) frequency acceleration and posterior or frontal alpha dynamics.

To assess whether inter-individual variability in session-long slopes was coordinated across brain regions, we examined participant-wise coupling of slope values across anatomically defined regions of interest across all datasets. This analysis revealed systematic inter-regional associations rather than a uniform shift across the scalp (Fig 5), consistently observed across all datasets. Specifically, greater mu frequency acceleration in central regions was associated with stronger posterior alpha slowing across participants (r = −0.02 to −0.18; predominantly small effects; Fig 5a). In parallel, stronger centroparietal mu acceleration was linked to frontal alpha slowing (r = −0.17 to −0.26; small-to-moderate effect sizes), while also showing a positive association with posterior alpha frequency acceleration with effects ranging from negligible to moderate depending on the dataset. Similar coupling patterns were observed for alpha/mu magnitude (Fig 5b), where associations were generally stronger (r = −0.43 to 0.47), corresponding to moderate effects and, in some datasets, approaching large effect sizes. These findings indicate coordinated changes in both oscillatory strength and temporal organization.

**Fig 6:**
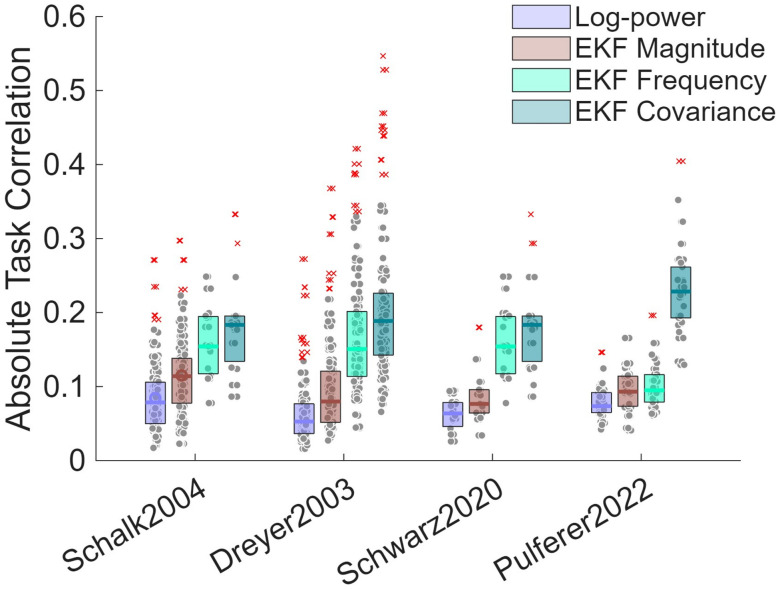
Comparison of absolute task correlations across spectral feature representations and datasets. Boxplots summarize the distribution of subject-wise maximum absolute Pearson correlations between task reference signals and four EEG feature types: EKF-tracked alpha/mu frequency, EKF-tracked alpha/mu magnitude, the Frobenius norm of the EKF state covariance matrix (reflecting estimator uncertainty and dynamical stability), and conventional alpha/mu log-power, computed within the same dataset-specific frequency range used for EKF tracking by squaring and applying a base-10 logarithm to the bandpass-filtered signal. For each subject and feature, correlations were computed separately for all channels and the maximum absolute value across channels was retained to provide a single representative measure per participant. Results are shown for all four datasets, demonstrating that EKF-derived features exhibit consistently stronger task correlations than fixed-band log-power features (*p* < 0.05 after correction for multiple comparisons in all datasets).

Building on these slope-based characterizations of session-long dynamics, we finally assessed their functional relevance for BCI decoding by comparing task-related correlations obtained from conventional spectral features and EKF-derived features. The results of this comparison are summarized in Fig 6. For each dataset, log-power was computed within the mu frequency range identified as task-relevant, and EKF-based frequency and magnitude features were derived accordingly. Across all datasets, EKF-based frequency and magnitude features showed significantly stronger task correlations compared to those obtained using band-limited log-power. Interestingly, the Frobenius norm of the EKF state covariance matrix also exhibited robust task correlations, indicating that estimator uncertainty and dynamic stability convey behaviorally relevant information beyond conventional power-based measures.

**Fig 7:**
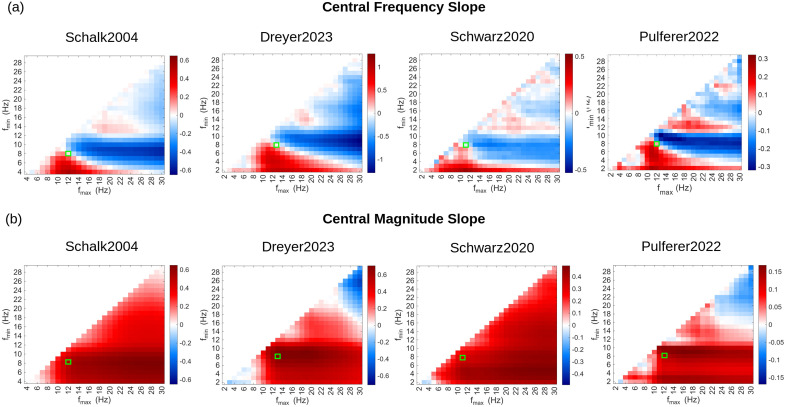
Slope maps of EKF-estimated (a) frequency (top row) and (b) magnitude (bottom row) changes across a wide range of bandpass filtered frequency bands, averaged over central EEG channels (i.e., Ci) and participants. Columns correspond to different datasets (Schalk2004, Dreyer2023, Schwarz2020 and Pulferer2022 from left to right). Each pixel represents the mean slope (in Hz/hour) for a specific band defined by [f_min_, f_max_]. Positive slopes (red) indicate rising frequencies, while negative slopes (blue) indicate decreasing trends. Green boxes highlight the frequency bands selected to generate the topographical maps of Fig 3.

Beyond the alpha/mu band, we next examined frequency-resolved slope trends across a broader spectral range. Fig 7a shows mu slope dynamics across the full 1–30 Hz range. Each pixel reflects the grand-average slope in central channels (Ci) for a given [f_min_, f_max_] range. Red indicates increasing frequency or magnitude over time; blue indicates decreases. Strong mu-band acceleration was seen in the 8–12 Hz range, while higher beta-band frequencies (~13–20 Hz) exhibited negative slopes, indicating beta slowing over central regions. Green boxes mark the dataset-optimized mu bands used in the main analysis (Fig 3), which interestingly lie at a critical spectral boundary where slope trends diverge: frequencies just below this range show global increases (Fig 7 focuses only on central channels), while frequencies above (i.e., in the beta range) exhibit spatially distinct decreases. Similarly, Fig 7b (central magnitude slopes) displays consistent patterns across all datasets, with widespread positive slopes, indicating a general increase in alpha/mu magnitude over time.

## Discussion

We investigated within-session changes in EEG alpha and mu rhythms during motor-related BCI tasks using an EKF-based oscillator tracking framework, applied to four public EEG datasets (Schalk2004, Dreyer2023, Schwarz2020, Pulferer2022). These included tasks from executed/imagined movements to naturalistic grasping. Across datasets, we consistently observed mu frequency increases over central motor areas, alpha slowing in surrounding areas, and rising alpha/mu magnitude that support and validate our initial hypothesis regarding training-induced spectral shifts. These divergent trends emerged within as little as ~15 minutes of BCI training (see Dreyer2023) and extended up to 50 minutes in longer sessions (see Pulferer2022).

### Mu acceleration over sensorimotor regions

Across datasets, mu frequency consistently increased over central sensorimotor regions, suggesting functional reorganization of motor networks during BCI calibration, potentially indicative of enhanced neural efficiency and engagement. Prior studies in motor learning and neurofeedback support this [26,27]. Importantly, Pfurtscheller [35] has shown that with training, the spatial extent of the upper mu (10–12 Hz) ERD tends to become more focused and topographically restricted. Such localized ERD may correspond to more functionally specific neural activation and could facilitate the emergence of faster, more coherent oscillatory activity in relevant motor areas. In this light, the observed mu frequency acceleration might not only reflect enhanced cortical excitability but also the result of spatial refinement and synchronization within trained motor networks. Topographical correlations between mu dynamics and task performance further support that these changes reflect true neural adaptations rather than artifacts. Importantly, this spatial distribution was closely reflected in the corresponding percentage maps (Fig 3a), which quantify the proportion of participants exhibiting significant positive slopes at each electrode. Channels displaying the strongest mu frequency increases were likewise those in which most participants showed positive slopes, suggesting that the central acceleration effect is not attributable to a small subset of individuals with large slope values, but instead represents a consistent pattern across subjects.

Despite common trends, dataset-specific differences were evident in both the spatial expression and consistency of mu acceleration, likely reflecting differences in task structure, session duration and movement modality. In Schalk2004, we observed bilateral increases in mu frequency and magnitude over central/centroparietal regions, consistent with its mix of unilateral and bilateral motor execution and imagery tasks, engaging both hemispheres. Topographical and task-correlation maps confirmed these effects as functionally meaningful. The Dreyer2023 dataset exhibited strong left-lateralized modulations. Although the Dreyer2023 protocol included both left- and right-hand motor imagery tasks for each subject, the group-level mu frequency modulation was more pronounced over left sensorimotor sites (C3). As the majority of participants in this dataset were right-handed, this asymmetry likely reflects common lateralization patterns associated with hand dominance, together with individual differences in imagery engagement and proficiency, which can bias group-level averages toward one hemisphere even when task conditions are balanced. The Schwarz2020 dataset showed a more diffuse but still central pattern of mu frequency modulation, likely due to its smaller sample size and bilateral grasp task, which may activate overlapping motor regions. Analysis of the Pulferer2022 dataset revealed comparatively more variable patterns, characterized by central frequency increases and less focused task correlations. This dataset differs substantially from the others in that movement was physically constrained and EEG was correlated with continuous kinematic measures rather than discrete task labels. These factors are likely to reduce the signal-to-noise ratio of trial-locked oscillatory modulation and emphasize slower, state-related dynamics, resulting in greater inter-individual variability and less spatially concentrated effects.

Importantly, slope maps estimated over a broader spectral range (Fig 7) revealed that the dataset-optimized mu bands used in the main analysis (Fig 3) coincided with a critical spectral boundary where slope trends diverged. At this operating point, between-subject variability in slope estimates was minimized, allowing spatial differences to emerge in the group average without being obscured by subject-specific baseline offsets. These offsets arise when the selected analysis band is misaligned with a participant’s dominant alpha/mu activity, biasing the estimated instantaneous frequency and magnitude toward a subject-specific spectral centroid. Because all electrodes within a subject share similar underlying spectral properties, this bias introduces a global shift that affects all channels in a similar manner (see Supporting Information: EEG Data – Effect of bandpass selection on alpha/mu frequency and magnitude slope topographies, S5, S6 and S7 Figs). As the analysis band moves away from this optimal range, spectral mismatch across participants increases, altering signal-to-noise ratio and slope scaling and inflating subject-dependent offsets that dominate group averaging and mask spatial structure. Although subject-wise mean removal can help recover spatial contrasts under these conditions, it also removes information about global frequency or magnitude changes, such as the broad alpha/mu magnitude increases observed across participants. Additional details are provided in the Supporting Information.

### Alpha/Mu magnitude increases over time

The global increase in alpha-band magnitude observed across the scalp during motor-related BCI calibration suggests a broad cortical shift over time. This effect extends beyond task-relevant regions, likely reflecting generalized neurophysiological processes such as cognitive fatigue. Käthner et al. [9] reported rising alpha power during prolonged P300-BCI use, coinciding with declining P300 amplitude, reduced performance, and increased self-reported fatigue. This supports the notion that rising alpha power is a reliable biomarker of cognitive fatigue [8]. Similar trends were found in SSVEP BCIs, where Cao et al. [36] noted alpha increases alongside reduced SSVEP amplitude and SNR. In pediatric BCIs, even 30 minutes of MI or P300 tasks led to elevated post-session alpha power [37]. In adults, sustained MI practice has shown mu/alpha power increases [38,39] even without immediate performance decline [39]. Benwell et al. [30] further demonstrated that alpha power increases and its peak frequency decreases systematically with continuous cognitive effort, reinforcing its role as a fatigue indicator.

Importantly, however, alpha and mu magnitude increases are probably not functionally equivalent across cortical regions. In central and centroparietal areas, increased mu-band magnitude likely reflects strengthening or stabilization of sensorimotor rhythms as participants adapt to the task and motor representations become more consistent. From a neurophysiological perspective, such increases in baseline mu power can be advantageous, as event-related desynchronization is defined relative to baseline activity [4]; a higher or more stable baseline mu amplitude can therefore support larger and more reliable ERD magnitudes during motor tasks, improving the contrast between rest and task states rather than indicating reduced engagement [40,41]. Importantly, this interpretation does not imply improved BCI decoding performance, as session-long baseline shifts can reduce classification accuracy unless appropriate normalization or adaptive classifier strategies are employed.

In contrast, magnitude increases observed outside sensorimotor regions (e.g., classical posterior or frontal alpha) are more plausibly linked to global state-related factors, such as fatigue, vigilance fluctuations [8,11], or reduced processing of task-irrelevant sensory input during prolonged calibration [3,42]. This distinction is particularly relevant for the Schalk2004 dataset, which involves repeated and prolonged motor execution and imagery tasks. In such settings, it is plausible that participants develop stronger and more stable sensorimotor rhythms over time (reflected in increased magnitude), while simultaneously exhibiting faster oscillatory dynamics (reflected in frequency acceleration) as sensorimotor representations become more refined. Although positive alpha/mu magnitude slopes are also visible over central and centroparietal regions in the other datasets, the strength and spatial specificity of the co-occurrence between magnitude increases and frequency acceleration appears most pronounced in Schalk2004, whereas in the remaining datasets the overlap is weaker or more spatially diffuse.

### Alpha slowing in non-sensorimotor regions

While sensorimotor regions showed mu frequency acceleration, posterior (parietal and occipital) and frontal areas, as well as surrounding non-sensorimotor areas (wherever applicable), consistently exhibited alpha slowing across datasets. This regional contrast suggests a shift in cortical resource allocation during BCI training. Alpha slowing has been linked to reduced vigilance, visual fatigue, and declining attention, often accompanied by increased alpha power [30], and is consistent with time-on-task and mental fatigue studies reporting systematic decreases in alpha peak frequency over posterior and frontal regions, alongside increases in low-frequency power, reflecting reduced alertness and disengagement from task-relevant processing [43,44].

However, rather than reflecting fatigue alone, this pattern may also indicate functional inhibition of task-irrelevant regions to support focused motor processing. As motor areas become more engaged, reflected in faster alpha/mu rhythms associated with improved motor readiness, non-sensorimotor regions may downregulate activity to enhance task-specific performance [42]. This interpretation is consistent with prior work showing that alpha peak frequency is a state-dependent and region-specific parameter, with cortical activation leading to alpha acceleration and reduced activation or input leading to alpha slowing [31]. Recent evidence further supports the notion that alpha-frequency organization is dynamically coordinated across regions: Smith et al. [45] demonstrated that peak-alpha frequency alternates between periods where it is relatively similar across the cortex and periods where it forms a clear posterior-to-anterior gradient, with slower alpha frequencies in frontal regions. Importantly, these changes are driven by distinct regional processes: posterior regions primarily influence the spatial pattern through changes in alpha power and synchronization, while anterior regions directly modulate alpha frequency. Together, these mechanisms account for a substantial portion of the observed variability in alpha frequency organization.

Our results are also consistent with this framework and extend it by showing that inter-individual variability in session-long frequency changes is systematically coupled across anatomically distinct regions. In all datasets, we found that participants with stronger mu frequency acceleration over central regions also exhibited more pronounced posterior alpha slowing. This inverse relationship fits with models proposing that alpha activity is generated by multiple interacting brain systems, rather than by a single global rhythm [11]. In practical terms, as individuals rely more on internally driven motor control, posterior regions involved in visual or sensory monitoring may become less dominant, expressed as a slowing of posterior alpha rhythms. On the other hand, mu acceleration over centroparietal regions was associated with frontal alpha slowing, potentially reflecting a reduced reliance on executive or attentional control as sensorimotor representations become more stable and automatized over the session [3]. At the same time, the positive association between centroparietal mu acceleration and posterior alpha frequency acceleration suggests that the centroparietal cortex may act as an interface linking sensorimotor and posterior networks. This is consistent with the role of centroparietal cortex in integrating sensory, motor, and attentional processes. In some individuals, alpha/mu rhythms across sensorimotor and posterior regions may become more temporally aligned as sensorimotor processing becomes more efficient. In other individuals, posterior regions may become less engaged in the task, reflected by a relative slowing of alpha-frequency activity. The fact that this coordination appears in both alpha/mu frequency and magnitude strengthens the argument that the observed effects reflect network-level adaptation. These interpretations, however, remain speculative and warrant further investigation.

Although the present data do not allow us to disentangle whether fatigue-related disengagement or functional inhibition primarily drives these effects, these mechanisms are likely not mutually exclusive and may jointly contribute to the observed pattern of alpha slowing, potentially with different relative contributions across individuals or over time.

### Broader spectral trends

While the main focus of this study was on alpha and mu rhythms, our exploratory analysis across a wider frequency range revealed additional trends of potential interest. Specifically, the frequency slope maps showed that, in central channels, beyond the alpha/mu range, higher frequencies such as those in the beta band (typically 13–30 Hz) predominantly exhibited negative frequency slopes over the course of BCI sessions, although positive trends were also observed depending on the selected analysis band (i.e., low beta vs high beta). Beta rhythms are linked to motor control, sensory integration, and sustained attention [46]. In this context, a decline in beta frequency over time may reflect a reduced dominance of fast, tightly controlled beta dynamics that are typically associated with tonic motor maintenance and sustained motor readiness [46]. Such changes may arise from alterations in motor readiness or attentional control related to task repetition, fatigue, or increasing automatization. Because mu and beta rhythms reflect complementary aspects of sensorimotor control, their dynamics can overlap spatially as these processes adapt in parallel, even though disentangling their individual contributions remains challenging.

### BCI implications

Overall, our findings show that alpha and mu rhythms evolve during BCI training in spatially and behaviorally meaningful ways. These dynamics have important implications for real-world BCI systems. Traditional BCI pipelines often assume fixed frequency bands, yet our results show that these rhythms can shift substantially even within a single session. If unaccounted for, such spectral shifts may degrade decoding accuracy over time, particularly in long or repeated-use scenarios [47]. This issue is particularly critical for spatial filtering methods like Common Spatial Patterns (CSP) [48], which extract discriminative features based on power differences in predefined frequency bands. This sensitivity of CSP to fixed spectral assumptions has also motivated earlier work on jointly optimizing spatial and spectral filters. Spatio-spectral extensions of CSP have been proposed to overcome fixed-band limitations, including time-delay embedding–based frequency adaptation by Lemm et al. [49] and combined spatio-temporal CSP optimization by Dornhege et al. [50], both demonstrating substantial performance gains through subject-specific spectral tuning.

In this context, several prior works have also explicitly emphasized the need to adapt spectral features over time to counter EEG non-stationarities. Thomas et al. [51] introduced an adaptive weighted spectral–spatial method that continuously updates the set of discriminative frequency components as a motor imagery session progresses. By explicitly addressing spectral non-stationarities, they demonstrated superior BCI performance compared to static-band approaches, underscoring the importance of tracking time-varying spectral structure. Similarly, here, our analysis further demonstrated that EKF-derived features, specifically tracked alpha/mu frequency, magnitude, and the EKF state covariance, exhibit task-related correlations that are significantly stronger than those obtained using conventional fixed-band log-power. This finding indicates that adaptive, model-based features capture behaviorally relevant information that may be missed by static power-based representations. In particular, the robust task correlations observed also for the EKF covariance feature suggest that estimator uncertainty and dynamical stability themselves carry functionally meaningful information, reinforcing earlier evidence that such quantities can support decoding in both motor execution and motor imagery paradigms. However, correlation alone is not the optimal metric for evaluating BCI performance. In practical BCI applications, decoding performance is more appropriately quantified using classification-oriented measures such as accuracy, F1 score, or Matthews correlation coefficient. Consequently, the true decoding performance of these features remains to be systematically evaluated.

In addition to improving decoding, tracking frequency and magnitude changes could inform the design of more effective BCI calibration paradigms. For example, rising mu frequency may indicate motor engagement or learning, while posterior alpha slowing can reflect fatigue or reduced attention. These markers can inform adaptive protocol changes, e.g., by adjusting task difficulty, adding breaks, or modifying feedback [52], to support performance and neuroplasticity. Such neuro-informed strategies may boost the efficiency and long-term sustainability of BCI training, especially in clinical or rehab settings.

It is worth noting here that the ability to detect such spectral changes may vary depending on the task modality. We hypothesize that mu acceleration is easier to observe during MI than ME. In ME, stronger ERD responses can significantly suppress mu power, potentially masking the oscillatory peak and complicating frequency tracking. MI, on the other hand, typically induces more moderate mu ERD [53] (refer to the supplementary material of [53], for an overview of ERD/S pattern variations by movement modality), preserving the mu rhythm’s structure and enabling more reliable estimation of its temporal dynamics. This hypothesis was not explicitly tested in the present study and is therefore raised as a potential confounding factor that may influence the detectability of session-long mu frequency changes across task modalities.

Another important point is that while group-level trends may also be detectable by averaging power spectral densities across participants, this approach can obscure individual differences due to spatial variability and inter-subject variation in mu peak frequency. Therefore, subject-specific tracking remains essential for accurately capturing the general dynamics of spectral adaptation.

These observations offer some food for thought, highlighting how task design and methodological choices may affect the detectability of training-related frequency shifts.

### Alpha/mu tracking framework

While our earlier cascade-form TV-AR method [33] was effective for capturing broadband spectral dynamics by estimating multiple poles, it lacked the ability to selectively isolate and track narrowband rhythms such as alpha and mu. This limitation made it challenging to study rhythm-specific changes in response to training. In contrast, the new method is explicitly optimized for narrowband oscillations by modeling frequency and damping as latent states within an EKF framework. This approach not only improves numerical stability and physiological interpretability but also enables precise tracking of moment-to-moment changes in frequency and amplitude. Crucially, the method is computationally efficient and can operate in real-time, making it well-suited for online BCI applications where adaptation to ongoing brain state dynamics is critical. Beyond BCI, this framework may also be useful in cognitive neuroscience, neurofeedback, and clinical monitoring, where robust and interpretable tracking of brain rhythms is essential.

### Limitations

While this study provides insights into within-session alpha/mu dynamics during motor-related BCI tasks, several limitations should be noted. First, accurately capturing spatially and spectrally specific effects, such as the divergence between mu acceleration and posterior alpha slowing, requires careful frequency band selection. We addressed this by integrating broad frequency slope maps with task-related correlations, but this approach may benefit from further refinement, such as data-driven or individualized frequency estimation in future work. Second, our tracking focused on offline calibration sessions; whether similar trends persist under online BCI feedback or in real-time settings remains an open question. Finally, the observed increase in alpha magnitude over time, especially across widespread scalp regions, may reflect a combination of neuroplastic changes, cognitive fatigue or declines in arousal. Disentangling these effects will require future studies with more controlled designs.

## Materials and methods

### Extended Kalman filter based oscillator tracking

To track time-varying frequency and magnitude in alpha and mu rhythms during BCI training, we employed an oscillator-based estimation approach grounded in a reparametrized second-order autoregressive (AR(2)) model. This model represents each EEG signal as the output of a damped linear oscillator, where the instantaneous frequency and damping factor evolve over time as latent variables. These latent dynamics were estimated using an extended Kalman Filter (EKF) applied to each bandpass filtered EEG channel, allowing continuous and noise-robust tracking of narrowband oscillatory activity. The EKF was implemented in a nonlinear state-space framework, with analytic Jacobians used to linearize the transition function at each step. To ensure accurate and stable tracking, key EKF hyperparameters, such as process and measurement noise covariances, initial frequency and damping values, and initial state uncertainty, were optimized using a genetic algorithm. Optimization was performed on the first 60 seconds of the EEG by minimizing a prediction error-based cost function averaged across all channels. Once optimal parameters were identified, they were applied to the full-length signals for tracking. To validate the method, we generated synthetic EEG signals with known time-varying frequency trajectories (Supporting Information, S1 and S2 Figs) and evaluated the EKF’s ability to recover ground truth dynamics. Performance was also compared against a standard Hilbert transform-based approach. Full model equations, optimization procedures and performance comparisons (S3 Fig) are provided in the Supporting Information.

### EEG dataset description

We applied alpha/mu-band EKF tracking to several publicly available EEG datasets involving motor-related tasks, all collected from healthy participants. We focused exclusively on offline calibration sessions, defined as the initial phase of a BCI experiment during which EEG is recorded while the participant performs predefined tasks (e.g., motor execution or motor imagery). These data are used to train and initialize the decoding models or feature-selection procedures before online or feedback-based BCI operation. We excluded any online runs to avoid confounds introduced by closed-loop or mutual human–machine adaptations. Specifically, we analysed: (i) the large-scale dataset by Schalk et al. [54] which involves unilateral and bilateral hand and foot movements under both real and imagined conditions; (ii) the Dreyer et al. [55] dataset featuring EEG recordings during left- and right-hand motor imagery following the Graz BCI protocol; (iii) the Schwarz et al. [56] dataset involving natural reach-and-grasp actions. EEG in the latter was recorded with gel‐based, water-based, and dry electrodes, however our analysis focused solely on data from the gel-based system; and (iv) the Pulferer et al. [57] dataset featuring a continuous target-tracking paradigm in which hand movements were largely suppressed by physical restraint. Together, these datasets provide a comprehensive testbed for alpha/mu tracking, capturing a variety of motor behaviors and recording conditions. A detailed description of the datasets can be found in Table 1.

### EEG preprocessing

All recordings were preprocessed using a standardized pipeline. Signals were first high-pass filtered at 0.5 Hz (4th-order Butterworth). Whenever necessary, notch filters were applied at 50/60 Hz and their harmonics to suppress power-line noise. To balance tracking performance with computational efficiency, based on insights from our simulations showing improved accuracy at higher sampling rates (see Supporting Information), all datasets were resampled to 120 Hz. Independent component analysis (ICA; extended Infomax [58] via EEGLAB [59]) was applied to remove non-neural artifacts, with components auto-labeled using ICLabel [60]. ICA results were manually reviewed and adjusted.

### Tracking alpha/mu dynamics

To assess training-related changes in alpha/mu rhythms, we analyzed only calibration sessions, excluding rest or artifact-removal blocks. Sessions were concatenated per participant to create continuous input for EKF tracking. While not strictly continuous in terms of cognitive engagement, this approximation enables a meaningful temporal mapping of how oscillatory dynamics evolve with BCI use. For Pulferer2022, sessions were analyzed separately due to multi-day recordings. Results were then averaged across sessions for each participant (i.e., 10 participants x 3 sessions).

For each dataset, we explored a limited set of candidate bandpass filter cutoffs by varying the lower and upper frequencies (f_min_, f_max_) between 6–8 Hz and 12–14 Hz, respectively, while keeping the sampling rate fixed at 120 Hz and the filter order fixed at 5. For each candidate frequency band, the EEG was first bandpass-filtered accordingly, and an EKF-based oscillator model was then applied to track instantaneous alpha/mu frequency and magnitude. For each bandpass-filtered signal, the EKF hyperparameters (including process noise, measurement noise, initial frequency, initial damping, and covariance scaling) were optimized using only the first 60 seconds of EEG data. This optimization was performed independently for each (f_min_, f_max_) pair to ensure that the EKF operated optimally under the corresponding spectral constraints. The resulting optimal EKF parameters were then applied to track alpha/mu dynamics over the full EEG recording for that band.

To isolate the task-relevant mu band and select the dataset-specific alpha/mu frequency band, we computed the absolute correlation between the EKF-estimated magnitude time series and the available task-related reference signals for each candidate (f_min_, f_max_) pair across all participants. These reference signals included binary task labels (e.g., ME/MI vs. rest for Schalk2004, left vs. right hand movement labels for Dreyer2023, lateral vs palmar grasp for Schwarz2020) or continuous kinematic variables (for Pulferer2022). Resulting task correlations were visualized topographically, revealing spatial patterns of modulation. The frequency band yielding the maximum absolute correlation was selected as the dataset-specific alpha/mu band and used for all subsequent analyses. This procedure ensures that both the bandpass filter and the EKF parameters are appropriately matched for each candidate band, while maintaining a clear separation between the optimization phase (restricted to the first 60 s) and the full-session tracking phase.

### Alpha/Mu slope change likelihood

To perform a sanity check for potential abrupt changes in alpha/mu activity within each session, we used MATLAB’s *findchangepts* function to detect the most prominent inflection point in each EKF-derived trajectory. This yielded a binary vector per channel, with ones marking slope change locations. These vectors were smoothed using a noncausal Gaussian kernel (3-minute variance) and summed across channels to produce a temporal histogram of concurrent slope changes. This aggregate was normalized to yield a slope change likelihood curve, indicating periods with elevated probability of abrupt spectral shifts. This procedure served as a control step, ensuring that the observed trends were sustained and monotonic, rather than driven by transient fluctuations.

### Topographical visualization of alpha/mu shifts

After confirming no major within-session discontinuities, we quantified how alpha/mu frequency and magnitude evolved over time by computing channel-wise slopes from EKF-derived trajectories using robust linear regression (MATLAB’s *robustfit* with bisquare weighting), with all trajectories z-scored prior to regression. This approach yields stable slope estimates (in Hz/hour or a.u./hour), minimizing outlier influence. Slopes surviving Benjamini–Hochberg correction [61] were averaged across participants and visualized as scalp maps to highlight consistent spatial patterns. In addition, we estimated electrode-wise percentage maps reflecting the proportion of participants exhibiting a statistically significant positive slope in either frequency or magnitude. The full analysis pipeline is shown in Fig 1.

### Estimation of session-long alpha/mu initial and final values

To facilitate interpretation of the estimated session-long trends, we derived initial and final alpha/mu frequency and magnitude values directly from the fitted robust linear regression models, rather than from windowed power spectral density estimates or instantaneous EKF-derived samples. Specifically, after estimating channel-wise session-long slopes using robust regression, the corresponding regression coefficients were used to predict oscillatory values at the beginning of the recording (t = 0) and at its end (t = T). This procedure yields stable estimates of starting and ending frequency and magnitude that are inherently consistent with the fitted session-long trajectory and are less sensitive to short-term fluctuations or local spectral variability. The resulting initial and final values were then aggregated across channels and participants within each dataset and visualized using scatter plots.

### Statistical assessment of spatial differences and coupling in alpha/mu slopes

To assess spatial differences in session-long alpha/mu dynamics, we performed a direct statistical comparison of slope magnitudes across EEG electrodes using paired tests across participants within each dataset. For each pair of electrodes (i,j), within-participant differences in slope values were computed, and a paired t-test was applied to evaluate whether slope magnitudes at electrode i differed significantly from those at electrode j. This analysis was conducted separately for alpha/mu frequency and magnitude slopes. Multiple comparisons across all electrode pairs were controlled using the Benjamini–Hochberg procedure. For statistically significant comparisons, paired-effect sizes were estimated using Cohen’s dz.

To also examine whether inter-individual variability in alpha/mu slopes was systematically coupled across brain regions, we performed a participant-wise correlation analysis across selected channel groups. EEG electrodes were grouped into four anatomically motivated regions of interest (ROIs): central, centroparietal, frontal, and posterior (parieto-occipital/occipital). Channel selections were chosen to be as consistent as possible across datasets to facilitate comparability. For each pair of electrodes, Pearson correlation coefficients were computed across participants using the corresponding slope values, yielding channel-by-channel correlation matrices for frequency and magnitude slopes. Statistical significance was assessed using two-sided tests, with correction for multiple comparisons across all channel pairs using the Benjamini–Hochberg procedure. Only correlations surviving correction were retained. Significant channel-wise correlations were subsequently averaged within and between ROIs to obtain reduced ROI-level correlation matrices summarizing inter-regional coupling of alpha/mu slope dynamics.

### Task relevance of EKF-derived alpha/mu features

In addition to using absolute task correlations to guide the selection of dataset-specific alpha/mu frequency bands for EKF tracking, we performed a complementary feature-level analysis to assess the practical relevance of session-long alpha/mu dynamics for BCI applications. For each dataset, we computed the absolute Pearson correlation between task reference signals and four feature types: (i) EKF-tracked alpha/mu frequency; (ii) EKF-tracked alpha/mu magnitude; (iii) the Frobenius norm of the EKF state covariance matrix, which reflects estimator uncertainty and dynamical stability and is obtained directly from the EKF recursive equations (Supporting Information, Eq. (8h)). This covariance-based feature has previously been shown by us to carry discriminative information for decoding motor execution and motor imagery tasks [33,62]. (iv) conventional alpha/mu log-power, computed within the same dataset-specific frequency range used for EKF tracking by squaring and applying a base-10 logarithm to the bandpass-filtered signal (no smoothing was applied).

Absolute correlations were estimated separately for each feature, channel, and subject. To obtain a single representative value per subject and feature, we selected the maximum absolute correlation across channels. These subject-wise maxima were then aggregated across participants within each dataset for group-level comparison. Statistically significant differences between features were assessed using the Wilcoxon signed-rank test, with p-values corrected for multiple comparisons using the Benjamini–Hochberg procedure.

### Frequency-resolved shift analysis in central channels

To further characterize the temporal dynamics of the motor cortex EEG activity across a range of frequency bands, we applied the EKF tracking approach to EEG signals bandpass filtered with 5th-order Butterworth filters. Frequency ranges were systematically defined using all valid combinations of lower and upper cutoff frequencies between 1 Hz and 30 Hz. For each frequency band, the EKF was used to extract time-varying estimates of instantaneous frequency and magnitude in central channels only (i.e., Ci). To assess whether these trajectories exhibited systematic trends over time, we applied robust linear regression to each channel’s time series and computed the slope estimates. This analysis was performed across all subjects. The resulting slope values were averaged (across channels and participants) to generate two summary matrices, one for frequency slopes and one for magnitude slopes, each capturing the direction and strength of temporal shifts across frequency bands.

## Supporting information

S1 Text Supporting Information. (PDF)

S1 Fig Representative simulated EEG time series and power spectral density (PSD) for three synthetic datasets. For each dataset (rows), the first ten (left column) and the last ten seconds (right column) of the multichannel signal are shown. Time-series plots display all channels overlaid, illustrating the broadband, noise-embedded oscillatory activity. Corresponding PSDs were estimated using Welch’s method (0.5-s Hanning window, 85% overlap) and show the combined theta, alpha, and beta components embedded in a 1/f-like background. Differences between the first and last segments reflect the imposed session-long changes in oscillatory frequency and magnitude.
(TIF)

S2 Fig Example time-domain decomposition of the simulated EEG signal for three representative channels. For each channel, the left column shows the first second of the simulations, and the right column shows the last second. The black trace denotes the final synthetic EEG signal, constructed as a weighted sum of band-limited components: pink noise (orange), theta (purple), alpha/mu (teal), and beta (red) (as in Eq. (11) of the Supporting Information).
(TIF)

S3 Fig Simulations – (a) Boxplots of Pearson correlation coefficients between estimated and ground truth slope values for alpha/mu-band frequency and magnitude across all EEG channels of the 20 synthetic datasets, comparing the EKF-based method with the Hilbert transform (HT). Each boxplot summarizes results across all tested preprocessing configurations, including variations in bandpass filter order, cutoff frequencies (fₘᵢₙ and fₘₐₓ), resampling rates, and the presence or absence of channel-wise standardization. Statistically significant differences are denoted with asterisks (***p < 0.001). (b) EEG topographical maps of alpha/mu-band frequency and magnitude slopes, visualizing spatial trends in ground truth, EKF-based estimates, and HT estimates from one representative synthetic EEG dataset. Red hues indicate increasing slopes (i.e., rising frequency or magnitude over time), while blue hues indicate decreasing slopes. The top row displays alpha/mu frequency slope (Hz/hour) distributions, and the bottom row displays magnitude slope (a.u./hour) distributions. Data were bandpass filtered between [6 14] Hz using a 5th-order Butterworth filter at 120 Hz sampling rate, with no standardization. The EKF method more accurately reproduced ground truth patterns than HT, with frequency slope correlations of 0.826 (EKF) vs. 0.792 (HT), and magnitude slope correlations of 0.935 (EKF) vs. 0.914 (HT).
(TIF)

S4 Fig Paired effect-size matrices comparing session-long slopes across electrodes, computed across participants. Each cell represents the paired difference in slope magnitude between a reference channel (j, x-axis) and a compared channel (i, y-axis). Red values indicate that slopes at channel i are larger than at channel j, whereas blue values indicate the opposite. Only statistically significant comparisons after Benjamini–Hochberg FDR correction are shown. The light-grey shaded area denotes electrodes over central and centroparietal regions. (a) Frequency slope comparisons. The strongest positive differences are centered over centroparietal electrodes, which exhibit significantly larger frequency increases than both frontal and posterior regions, while central electrodes differ mainly from frontal sites. (b) Magnitude slope comparisons. Both central and centroparietal electrodes show significantly larger magnitude increases than frontal regions. Compared to posterior regions, centroparietal sites exhibit consistently larger magnitude slopes, whereas central electrodes show weaker or channel-specific differences.
(TIF)

S5 Fig Topographical maps show slopes of EKF-estimated alpha/mu frequency (top rows) and magnitude (bottom rows) across four candidate bandpass ranges ([7–12], [7–13], [8–12], and [8–13] Hz). Panel (a) shows slopes computed from raw (non-normalized) trajectories, whereas panel (b) shows slopes after subject-wise mean removal to isolate spatial contrasts. Red colors indicate increases over the session, and blue colors indicate decreases. The Fig illustrates that bandpass selection strongly affects the global offset of estimated slopes: bands slightly below the dominant alpha/mu peak yield globally positive frequency slopes, while bands above the peak yield globally negative slopes. Importantly, the underlying spatial structure, characterized by central/centroparietal frequency increases and posterior/frontal slowing, remains preserved across bands and becomes more apparent after mean removal. In contrast, alpha/mu magnitude exhibits robust, spatially consistent increases across all tested bands, indicating broad session-long amplitude growth that is less sensitive to bandpass choice. Panel (b) however, depicts the spatial pattern after this global growth has been removed.
(TIF)

S6 Fig Topographical maps show slopes of HT-estimated alpha/mu frequency (top rows) and magnitude (bottom rows) across four candidate bandpass ranges ([7–12], [7–13], [8–12], and [8–13] Hz). Panel (a) shows slopes computed from raw (non-normalized) trajectories, whereas panel (b) shows slopes after subject-wise mean removal to isolate spatial contrasts. Red colors indicate increases over the session, and blue colors indicate decreases. The Fig illustrates that bandpass selection strongly affects the global offset of estimated slopes: bands slightly below the dominant alpha/mu peak yield globally positive frequency slopes, while bands above the peak yield globally negative slopes. Importantly, the underlying spatial structure, characterized by central/centroparietal frequency increases and posterior/frontal slowing, remains preserved across bands and becomes more apparent after mean removal. In contrast, alpha/mu magnitude exhibits robust, spatially consistent increases across all tested bands, indicating broad session-long amplitude growth that is less sensitive to bandpass choice. Panel (b) however, depicts the spatial pattern after this global growth has been removed.
(TIF)

S7 Fig PSD-based validation of alpha/mu magnitude and frequency changes (Schalk2004 dataset). Topographical maps show differences between the last 5 minutes and the first 5 minutes (last – first) of the recording, computed using Welch’s power spectral density estimation. Left: change in alpha/mu-band power, obtained by extracting peak alpha/mu power within the dataset-specific band (i.e., [8 12] Hz for Schalk2004) for each electrode. Right: change in alpha/mu peak frequency, extracted from the PSD within the same band.
(TIF)
