# Supplementary material for: Opposing cortical forces: Alpha slowing and sensorimotor mu acceleration during motor-related BCI training
Source: PLoS Comput Biol. 2026 Apr 1;22(4):e1014112. doi: 10.1371/journal.pcbi.1014112 (PMC13065009; doi:10.1371/journal.pcbi.1014112)
Supplement: S1 Text — (PDF) [file pcbi.1014112.s008.pdf]

Supporting Information for

Opposing Cortical Forces: Alpha Slowing and Sensorimotor Mu Acceleration during Motor-Related BCI Training

Alpha slowing and mu acceleration during motor-related BCI training

Kyriaki Kostoglou[1], Gernot R. Müller-Putz[1,2]

[1]Institute of Neural Engineering, Graz University of Technology, Graz, Austria

[2]BioTechMed, Graz, Austria

*Gernot R. Müller-Putz.

Email: gernot.mueller@tugraz.at

Supporting information description

This appendix details the Kalman-based oscillator tracking framework used to estimate time-varying alpha/mu frequency and magnitude in EEG signals. It includes a full mathematical derivation of the reparametrized second-order autoregressive (AR(2)) model, its state-space formulation, and the implementation of the extended Kalman Filter (EKF), including analytic expressions for the Jacobian and update equations. We also describe the hyperparameter optimization procedure using a genetic algorithm, outlining the cost function and initialization strategy. Finally, we validate the method's ability to track frequency/magnitude changes through a simulation study using synthetic EEG data with known oscillatory trajectories. This appendix further includes additional EEG data analyses not shown in the main manuscript.

Kalman filter-based oscillator tracking

To capture instantaneous modulations in alpha and mu rhythms, we implemented an extended Kalman Filter (EKF) based on a time-varying second-order autoregressive (AR(2)) resonator model. Each EEG signal was first bandpass filtered to isolate the target frequency range, ensuring that the tracked latent oscillator remained within physiologically plausible bounds and minimizing the influence of unrelated spectral components. The filtered signals were then modeled as the output of a linear oscillator driven by stochastic excitation [1–3].

In this framework, the standard AR(2) model expresses each EEG sample as a linear combination of its two preceding values and a stochastic input term [4,5]:

$$x(n) = a_1(n)x(n-1) + a_2(n)x(n-2) + v(n) \tag{1}$$

This model can exhibit oscillatory behavior when the roots of its characteristic equation

$$\zeta^2 - a_1(n)\zeta - a_2(n) = 0 \tag{2}$$

form a pair of complex conjugates of the form $\zeta_{1,2} = re^{\pm j\theta}$ where $r \in [0\ 1]$ controls the damping (decay) and θ defines the angular frequency, mirroring the behavior of a discrete-time harmonic oscillator [6]. If a quadratic has roots $\zeta_{1,2}$, its factorized form is:

$$(\zeta - \zeta_1)(\zeta - \zeta_2) = (\zeta - re^{j\theta})(\zeta - re^{-j\theta}) = \zeta^2 - 2rcos(\theta)\zeta + r^2 \quad (3)$$

Thus $a_1(n) = 2rcos(\theta)$ and $a_2(n) = -r^2$. To make the model parameters physiologically interpretable, we reparametrized the AR coefficients $a_1(n)$ and $a_2(n)$ as functions of the instantaneous frequency ω_n (in radians/sample) and damping coefficient α_n (in s^{-1}) of the oscillator. Assuming the pole magnitude and angle are related to these parameters as $r = e^{-\alpha_n T_s}$ and $\theta = \omega_n T_s$, where $T_s = \frac{1}{F_s}$ is the sampling interval, then the AR coefficients become $a_1(n) = 2e^{-\alpha_n T_s}\cos(\omega_n T_s)$ and $a_2(n) = -e^{-2\alpha_n T_s}$ (by considering the continuous-time solution of a damped harmonic oscillator). Substituting into Eq.(1) yields the reparametrized oscillator model,

$$x(n) = 2e^{-\alpha_n T_s}\cos(\omega_n T_s)\, x(n-1) - e^{-2\alpha_n T_s} x(n-2) + v(n) \quad (4)$$

The term $v(n) \sim N(0, \boldsymbol{Q})$ denotes process noise with $\boldsymbol{Q} = Q_{ct} T_s \boldsymbol{I_4}$ representing the process noise covariance scaled to discrete time. In essence, Eq.(4) is an AR(2) model reparametrized to reflect a damped harmonic oscillator, whose coefficients are tied to magnitude and frequency parameters. Although our AR(2) model operates entirely in discrete time, we reparametrized the pole magnitude and angle in terms of continuous-time damping α_n and angular frequency ω_n to enhance physiological interpretability and cross-dataset comparability. Continuous-time parameters are independent of sampling rate, align more naturally with physiological units (e.g., Hz, decay per second), and connect to established continuous-time models of neural dynamics.

To facilitate tracking of the latent frequency and magnitude dynamics, the oscillator is expressed as a state-space model using two internal resonator states $x_1(n)$ and $x_2(n)$, along with the time-varying frequency and damping parameters,

$$x_1(n) = 2e^{-\alpha_n T_s}\cos(\omega_n T_s)\, x_1(n-1) - e^{-2\alpha_n T_s} x_2(n-1) + v(n) \quad (5a)$$

$$x_2(n) = x_1(n-1) \quad (5b)$$

$$\omega_n = \omega_{n-1} \quad (5c)$$

$$\alpha_n = \alpha_{n-1} \tag{5d}$$

These equations define the nonlinear transition model for the extended state vector $\boldsymbol{z}(n) = [x_1(n) \quad x_2(n) \quad \omega_n \quad \alpha_n]^T$ which evolves recursively. The observation model is linear, assuming the measured signal $y(n)$ arises from the first oscillator component plus Gaussian measurement noise,

$$y(n) = \boldsymbol{Hz}(n) + \varepsilon(n), \;\; \boldsymbol{H} = [1 \quad 0 \quad 0 \quad 0] \tag{6}$$

The noise term $\varepsilon(n) \sim N(0, R)$ represents measurement noise with variance scaled to the sampling interval as $R = R_{ct}T_s$. The EKF performs recursive estimation by linearizing the nonlinear state-transition function around the current estimate. At each step, the Jacobian of the transition function $\boldsymbol{F}_n = \frac{\partial f}{\partial \boldsymbol{z}}$ is computed analytically as,

$$\boldsymbol{F}_n = \frac{\partial f}{\partial \boldsymbol{z}} = \begin{bmatrix} \frac{\partial x_1(n)}{\partial x_1(n-1)} & \frac{\partial x_1(n)}{\partial x_2(n-1)} & \frac{\partial x_1(n)}{\partial \omega_n} & \frac{\partial x_1(n)}{\partial \alpha_n} \\ 1 & 0 & 0 & 0 \\ 0 & 0 & 1 & 0 \\ 0 & 0 & 0 & 1 \end{bmatrix} \tag{7a}$$

$$\frac{\partial x_1(n)}{\partial x_1(n-1)} = 2e^{-\alpha_n T_s} \cos(\omega_n T_s) \tag{7b}$$

$$\frac{\partial x_1(n)}{\partial x_2(n-1)} = -e^{-2\alpha_n T_s} \tag{7c}$$

$$\frac{\partial x_1(n)}{\partial \omega_n} = -2T_s e^{-\alpha_n T_s} \sin(\omega_n T_s)\, x_1(n-1) \tag{7d}$$

$$\frac{\partial x_1(n)}{\partial \alpha_n} = -2T_s e^{-\alpha_n T_s} \cos(\omega_n T_s)\, x_1(n-1) + 2T_s e^{-2\alpha_n T_s} x_2(n-1) \tag{7e}$$

Using this Jacobian, the EKF recursively applies the standard prediction and correction equations as follows [7],

Prediction:

$$\hat{\boldsymbol{z}}(n) = f\{\hat{\boldsymbol{z}}(n-1)\} \tag{8a}$$

$$\boldsymbol{P}(n) = \boldsymbol{F}_n \boldsymbol{P}(n-1) \boldsymbol{F}_n^T + \boldsymbol{Q} \tag{8b}$$

Update:

$$\hat{y}(n) = \boldsymbol{H}\hat{\boldsymbol{z}}(n) \tag{8c}$$

$$\hat{\varepsilon}(n) = y(n) - \hat{y}(n) \tag{8d}$$

$$S(n) = \boldsymbol{H}\boldsymbol{P}(n)\boldsymbol{H}^T + R \tag{8e}$$

$$\boldsymbol{K}(n) = \boldsymbol{P}(n)\boldsymbol{H}^T S(n)^{-1} \tag{8f}$$

$$\hat{\boldsymbol{z}}(n) = \hat{\boldsymbol{z}}(n) + \boldsymbol{K}(n)\hat{\varepsilon}(n) \tag{8g}$$

$$\boldsymbol{P}(n) = (\boldsymbol{I} - \boldsymbol{K}(n)\boldsymbol{H})\boldsymbol{P}(n) \tag{8h}$$

where $\boldsymbol{P}(n)$ is initialized with $\boldsymbol{P}(0)= P_0\boldsymbol{I}$ and $\hat{\boldsymbol{z}}(0) = [0 \quad 0 \quad \omega_0 \quad \alpha_0]^T$. The frequency is wrapped at each step to the fundamental Nyquist interval. Finally, the instantaneous frequency (in Hz) and magnitude are extracted from the posterior state estimates as,

$$f_n = \frac{|\omega_n|}{2\pi}, \qquad A_n = \sqrt{x_1(n)^2 + x_2(n)^2} \tag{9}$$

To robustly estimate the latent frequency dynamics, we optimized the hyperparameters of the EKF model using a data-driven approach based on a genetic algorithm (GA) [8–10]. The optimization targeted the parameter vector $\boldsymbol{X} = [R_{ct}\ Q_{ct}\ \omega_0\ \alpha_0\ \ P_0]$ comprising the measurement noise intensity, process noise intensity, initial angular frequency, initial damping coefficient, and the initial covariance scaling. To ensure numerical stability and physiological plausibility, all parameters were constrained within predefined bounds; in particular, the lower bounds were set to 1×10^{-7}, and the upper bounds to infinity. The GA was configured with a hybrid strategy combining global search and local refinement (via MATLAB's function *fmincon*) to minimize a prediction error-based cost function. The optimization was performed using only the first 60 seconds of the data per channel, ensuring a fixed-length and stable segment for evaluating candidate parameter sets. For each candidate $\boldsymbol{X}$, the EKF tracking was applied to the training segment, and the resulting normalized prediction error was averaged across channels to define the cost. Once the optimal parameter set $\boldsymbol{X}$ was obtained, the EKF was re-applied using these parameters on the full-length signal, allowing for consistent and interpretable tracking throughout the remaining data.

Simulations

Synthetic signals

To validate our tracking algorithm under controlled conditions, we generated 20 synthetic EEG-like datasets using AR processes with time-varying spectral properties. Each dataset comprised 38 spatial channels ('F5','F3','F1','Fz','F2','F4','F6','FC5','FC3','FC1','FCz','FC2','FC4','FC6','C5','C3','C1','Cz','C2','C4','C6','CP5','CP3','CP1','CPz','CP2','CP4','CP6','P5','P3','P1','Pz','P2','P4','P6','O1','Oz','O2') and simulated 20 minutes of brain activity sampled at 500 Hz. The signals included distinct oscillatory components in the theta (4–7 Hz), alpha/mu (8–12 Hz), and beta (13–30 Hz) bands, each embedded within colored pink noise to approximate realistic neural background activity. For each channel, pink noise was synthesized by passing zero-mean Gaussian white noise through a 4th-order Butterworth filter that approximates a 1/f spectral profile. To emulate the spectral characteristics of EEG, this pink noise was then band-limited between 0.5 and 70 Hz using a zero-phase Butterworth bandpass filter. Three distinct oscillatory processes: theta, alpha/mu and beta were synthesized using AR(2) models with time-varying pole parameters. Each AR component followed a resonator model defined by the recurrence:

$$\psi(n) = 2r_n \cos(w_n)\, \psi(n-1) - r_n^2 \psi(n-2) + e(n) \tag{10}$$

where r_n is the time-varying pole magnitude, $w_n = \frac{2\pi f_n}{F_s}$ the normalized angular frequency, and $e(n)$ is band-specific white noise. Each oscillation was generated independently per channel, with variations in frequency and magnitude introduced to simulate realistic intra- and inter-channel heterogeneity.

- *Theta Band*: Each channel was randomly assigned one of three temporal patterns: constant frequency, increasing frequency, or decreasing frequency. Corresponding pole magnitudes were varied in parallel.

- *Alpha/Mu Band*: Roughly one-third of the channels maintained constant alpha frequencies, while the remainder exhibited linearly increasing or decreasing frequency and damping patterns. The direction of change was spatially biased: anterior (i.e., frontal and central) channels tended to exhibit increasing frequencies and growing magnitudes, and posterior channels decreasing frequencies and increasing damping.
- *Beta Band*: Central channels were simulated with increasing beta frequency and decreasing magnitude (i.e., linearly fading power over time), while parietal and occipital channels showed the opposite trend. Channels not located in these regions were assigned constant beta parameters drawn from uniform distributions.

Small stochastic perturbations were applied to both frequency and magnitude trajectories to mimic natural fluctuations. Amplitude variability for each oscillatory component (theta, alpha/mu, beta) was introduced by scaling the driving white noise of the AR process. Specifically, band-specific global amplitude scalars were drawn from uniform distributions for each channel. The final EEG signal for each channel was constructed as a weighted sum of its components (Fig. S1),

$$EEG_{ch}(n) = 0.5 * Pink_{ch}(n) + 1.0 * Theta_{ch}(n) + 0.7 * Alpha_{ch}(n) + 0.5 * Beta_{ch}(n) \quad (11)$$

The weights used to combine pink noise with theta, alpha, and beta oscillatory components in the synthetic EEG signals (Eq.(11)) were determined empirically. They were selected through iterative testing and visual inspection of the resulting power spectra to ensure that the composite signals exhibited realistic EEG-like spectral characteristics. Specifically, pink noise was included at a moderate level (0.5) to simulate the 1/f background commonly observed in EEG. The theta component was given the highest weight (1.0) to ensure prominent low-frequency activity, while the alpha component, being the primary focus of the tracking analysis, was emphasized with a strong but slightly lower weight (0.7) to prevent it from dominating the spectrum. Beta oscillations were included with a modest weight (0.5) to introduce higher-frequency content typically present in EEG without overpowering the lower bands. Representative examples of the simulated EEG time series are shown in Fig. S1, while Fig. S2 illustrates example decompositions of the synthetic signals into their oscillatory components.

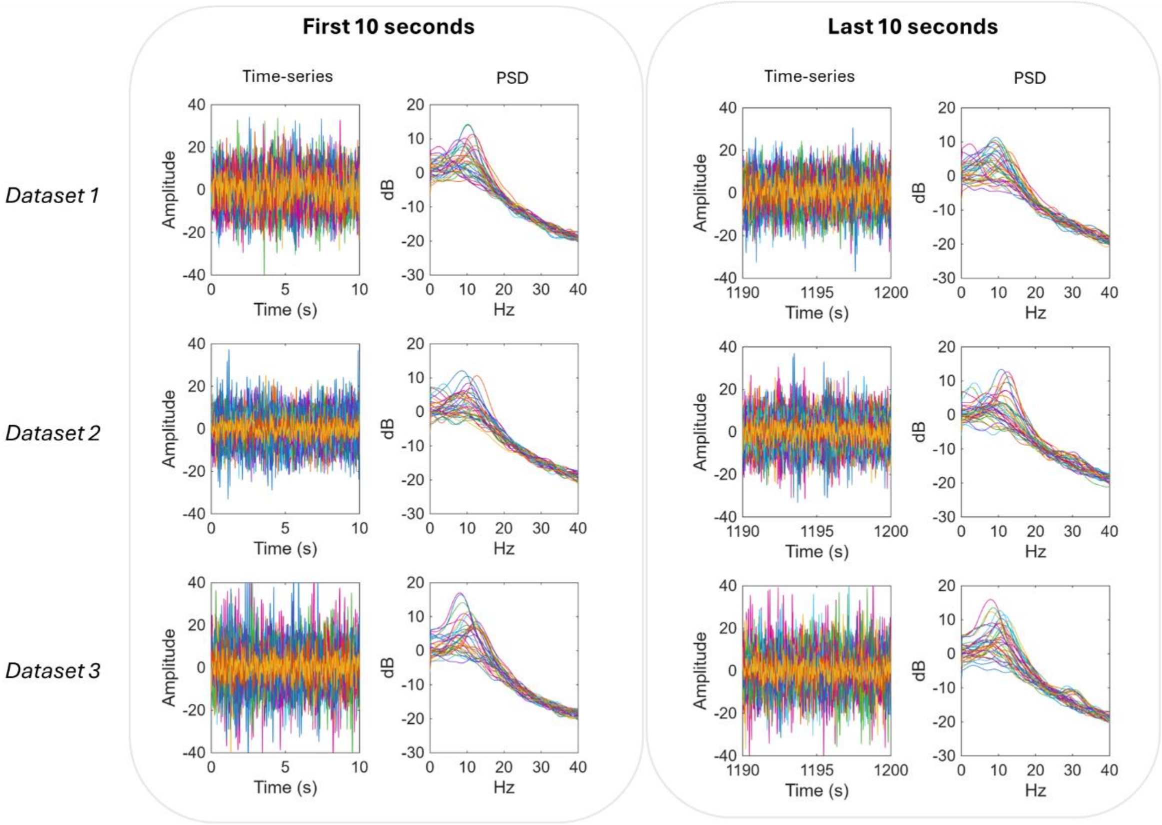

Figure S1. Representative simulated EEG time series and power spectral density (PSD) for three synthetic datasets. For each dataset (rows), the first ten (left column) and the last ten seconds (right column) of the multichannel signal are shown. Time-series plots display all channels overlaid, illustrating the broadband, noise-embedded oscillatory activity. Corresponding PSDs were estimated using Welch's method (0.5-s Hanning window, 85% overlap) and show the combined theta, alpha, and beta components embedded in a 1/f-like background. Differences between the first and last segments reflect the imposed session-long changes in oscillatory frequency and magnitude.

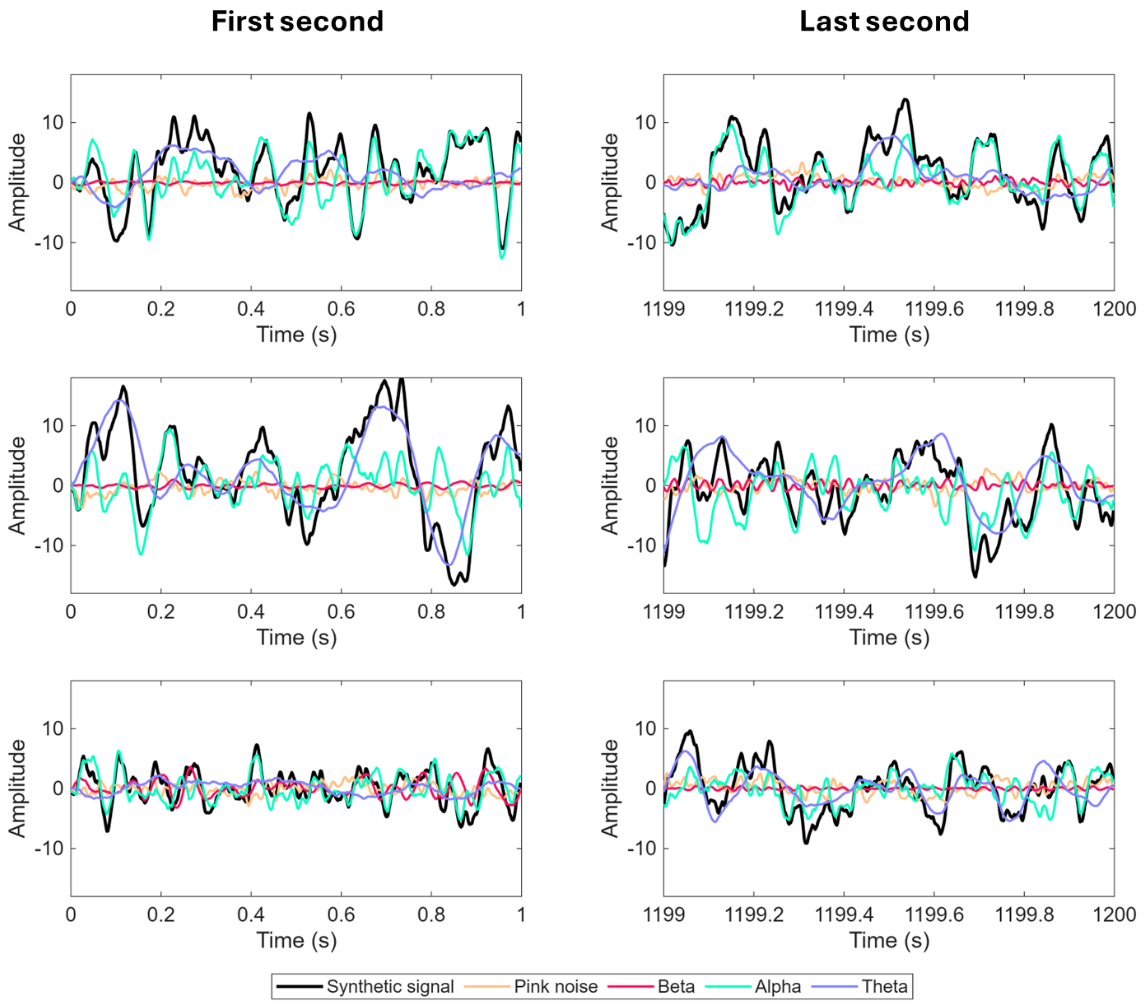

Figure S2. Example time-domain decomposition of the simulated EEG signal for three representative channels. For each channel, the left column shows the first second of the simulations, and the right column shows the last second. The black trace denotes the final synthetic EEG signal, constructed as a weighted sum of band-limited components: pink noise (orange), theta (purple), alpha/mu (teal), and beta (red) (as in Eq.(11)).

Tracking alpha/mu dynamics

Although the synthetic EEG signals contain embedded theta, alpha/mu, and beta oscillations to mimic realistic spectral complexity, the primary objective of this study was to accurately track only the alpha/mu-band dynamics. The inclusion of non-alpha components served solely to increase the ecological validity and difficulty of the estimation task, challenging the tracker to isolate alpha/mu activity in the presence of competing frequency peaks. To ensure that the Kalman-based

oscillator operated exclusively within the alpha/mu range, each signal was first preprocessed using a zero-phase Butterworth bandpass filter. The lower and upper cutoff frequencies were systematically varied across $f_{min} = \{6, 7, 8\}$ Hz and $f_{max} = \{12, 13, 14\}$ Hz, and the filter order was tested at values of 2, 3, 4, and 5 to examine tracking robustness under different frequency isolations. Following bandpass filtering, the signals were resampled to lower sampling rates (using MATLAB's resample function, which internally applies anti-aliasing filtering before interpolation). Resampling rates of $f_s = \{30, 60, 90, 120\}$ Hz were considered, reflecting the assumption that the target alpha/mu-band dynamics can be accurately tracked at reduced temporal resolutions. Additionally, we evaluated the effect of signal standardization, in which each EEG channel was z-scored by subtracting its mean and dividing by its standard deviation. This normalization step was intended to mitigate inter-channel amplitude variability and enhance stability during tracking.

Assessing temporal shifts in alpha/mu frequency and magnitude

To quantify how alpha/mu frequency and magnitude evolved over time, we computed the slope of their time courses on a channel-by-channel basis. Robust linear regression was applied to the EKF-derived trajectories using MATLAB's *robustfit* function with default settings. This method fits a linear model via iteratively reweighted least squares, employing a bisquare weighting function to reduce the influence of outliers by down-weighting data points with large residuals. This approach produces stable slope estimates even in the presence of non-Gaussian signal deviations or localized anomalies. The resulting slopes (in Hz/hour for frequency and arbitrary units/hour for magnitude) capture the rate of spectral change over time. Only slope values that survived multiple comparisons correction using the Benjamini-Hochberg procedure [11] were retained for further analysis. These significant values, computed across the scalp, were visualized using EEG topographical maps, offering a representation of the spatial distribution of temporal dynamics in alpha/mu activity.

Validation against ground truth

To quantitatively assess the accuracy of EKF-based oscillator tracking, we implemented a channel-wise evaluation framework comparing the estimated time-varying alpha/mu-band frequency and magnitude dynamics against ground truth trajectories derived from simulated EEG data. For each synthetic dataset and each EEG channel, the EKF-tracked instantaneous frequency and magnitude were extracted and compared to the known underlying frequency and magnitude profiles used during simulation. To establish an independent reference, we also applied the Hilbert transform (HT) [12] to the bandpass filtered signals to extract analytic estimates of instantaneous frequency and envelope. This method is widely used for empirical signal analysis and served as an additional benchmark for evaluating the fidelity of EKF tracking.

To achieve temporal alignment, the ground truth alpha/mu frequency and magnitude trajectories were resampled to match the respective analysis sampling rates (i.e., 30, 60, 90, or 120 Hz), allowing direct comparison between estimated and reference signals for each condition. For each channel, we then applied the same robust regression approach described earlier, enabling slope-based comparisons between estimated and true signals. Tracking performance was summarized by computing the Pearson correlation between estimated and true slopes across all channels for frequency and magnitude. These metrics quantify the EKF's ability to recover the temporal trends of underlying alpha/mu-band modulations on a per-channel basis, emphasizing fidelity to the dynamics rather than pointwise accuracy. To statistically compare the performance of the EKF method with the HT-based approach in the simulation studies, we employed nonparametric inference using the Wilcoxon signed-rank test. Effect sizes (based on Cohen's *r*) are reported where applicable to provide additional insight into the magnitude of observed differences.

Results

Figure S3a illustrates the Pearson correlation coefficients between the estimated and ground truth slope values for alpha/mu-band frequency and magnitude across all EEG channels, comparing the EKF-based method with the HT. The boxplots summarize results obtained across all tested preprocessing configurations, including variations in bandpass filter order, cutoff frequencies,

resampling rates and the presence or absence of channel-wise signal standardization. As the synthetic signals were generated using AR-based models, the simulation framework is inherently more closely aligned with the EKF-based oscillator model than with the HT approach, and may therefore favor EKF performance. Nevertheless, the purpose of this comparison was not to optimize performance for either method, but to assess whether the EKF yields frequency and magnitude slope estimates that are consistent with, and close to, those obtained using the HT under a wide range of preprocessing conditions. Within this context, the EKF approach consistently outperformed the HT method for both frequency ($p < 0.05$, $r = 0.622$) and magnitude ($p < 0.05$, $r = 0.330$) slope estimation. Interestingly, the tracking of magnitude slopes yielded higher overall correlations than frequency, suggesting greater robustness of magnitude dynamics to noise and preprocessing variability.

To assess the influence of preprocessing parameters on the EKF tracking performance, we fitted a linear model with the correlation between estimated and true alpha/mu-band frequency/magnitude slopes as the dependent variable and the bandpass filter cutoffs (f_{min}, f_{max}), resampling rate (f_s), filter order and standardization as predictors. For tracking, f_{min}, f_{max}, and f_s showed statistically significant effects ($p < 0.05$; $r = 0.443$, $r = 0.197$, $r = 0.08$, respectively, for frequency tracking and $r = 0.589$, $r = 0.556$, $r = 0.313$, respectively, for magnitude tracking). Filter order had a minimal yet statistically significant positive effect ($r = 0.035$ for frequency tracking and $r = 0.039$ for magnitude tracking). Standardization had no observable impact. Comparable patterns were found for HT-based tracking. These results underscore the importance of carefully selecting bandpass filter characteristics when applying the EKF tracking.

For visualization purposes in Figure S3b, we depict topographical maps of the alpha/mu frequency and magnitude slopes, ground truth and estimated ones from EKF and HT for one representative synthetic EEG dataset. In this simulation, both the EKF-based and HT-based methods were evaluated using identical preprocessing parameters: [6 14] Hz bandpass filter (5th-order Butterworth), applied to data sampled at 120 Hz, with no standardization. The goal was to assess each method’s ability to recover the ground-truth temporal slope patterns of frequency and magnitude embedded in synthetic signals. The HT-based method achieved a Pearson correlation

of 0.792 for frequency slopes and 0.914 for magnitude slopes against ground truth. In comparison, the EKF-based method yielded higher correlations of 0.826 for frequency and 0.935 for magnitude, demonstrating improved tracking accuracy for both oscillatory features.

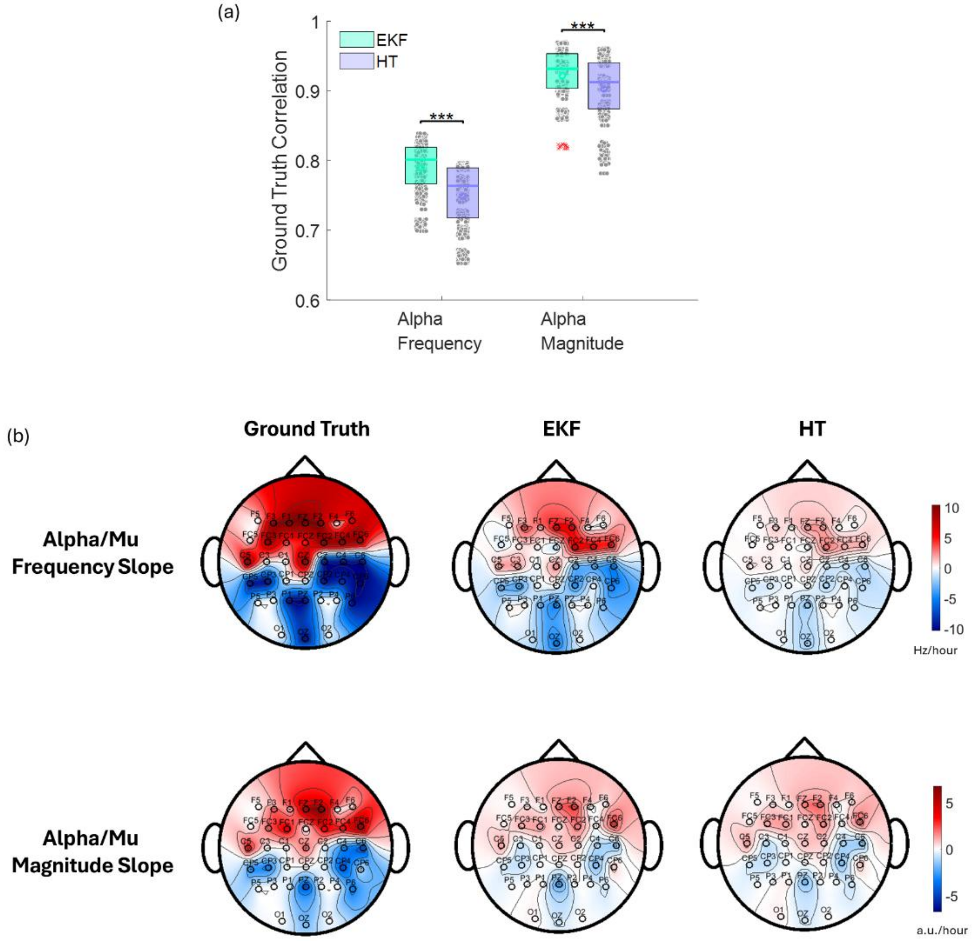

Figure S3. Simulations – (a) Boxplots of Pearson correlation coefficients between estimated and ground truth slope values for alpha/mu-band frequency and magnitude across all EEG channels of the 20 synthetic datasets, comparing the EKF-based method with the HT. Each boxplot summarizes results across all tested preprocessing configurations, including variations in bandpass filter order, cutoff frequencies (f_{min} and f_{max}), resampling rates, and the presence or absence of channel-wise standardization. Statistically significant differences are denoted with asterisks (***$p < 0.001$). (b) EEG topographical maps of alpha/mu-band frequency and magnitude slopes, visualizing spatial trends in ground truth, EKF-based estimates, and HT estimates from one representative synthetic EEG dataset. Red hues indicate increasing slopes (i.e., rising frequency or magnitude over time), while blue hues indicate decreasing slopes. The top row displays alpha/mu frequency slope (Hz/hour) distributions, and the bottom row displays magnitude slope (a.u./hour) distributions. Data were bandpass filtered between [6 14] Hz using a 5th-order Butterworth filter at 120 Hz sampling rate, with no standardization. The EKF method more accurately reproduced ground truth patterns than HT, with frequency slope correlations of 0.826 (EKF) vs. 0.792 (HT), and magnitude slope correlations of 0.935 (EKF) vs. 0.914 (HT).

EEG data

This section presents additional analyses of the EEG data that complement the results reported in the main manuscript. These analyses were not included in the main text but are provided here to support the interpretation and robustness of the reported findings.

Pairwise inter-channel slope comparisons

We performed a direct statistical comparison of slope magnitudes across electrodes using paired tests across participants. Specifically, for each pair of electrodes *(i,j)* we computed the within-participant difference in slopes and applied a paired t-test to assess whether slopes at electrode *i* were significantly different from those at electrode *j*. We controlled for multiple comparisons across all electrode pairs using Benjamini–Hochberg FDR correction, and we estimated the corresponding paired-effect sizes (Cohen's *dz*) for statistically significant comparisons. Statistically significant inter-electrode differences were observed only in the Schalk2004 dataset, whereas no effects survived correction in the remaining datasets. Figure S4 illustrates pairwise statistical comparisons of alpha/mu frequency and magnitude slopes across EEG electrodes for the Schalk2004 dataset. Panel (a) shows paired effect sizes for frequency slopes, while panel (b) shows the corresponding results for magnitude slopes. Each matrix entry represents the within-participant difference in slope between a pair of electrodes, with red values indicating significantly larger slopes at the compared electrode (*i*) than at the reference electrode (*j*), and blue values indicating the opposite (FDR-corrected across all electrode pairs).

Frequency slope comparisons (Fig. S4a):

- The strongest and most consistent effects are localized over centroparietal electrodes, which exhibit significantly larger positive frequency slopes than both frontal and posterior (parietooccipital and occipital) regions.
- Central electrodes show significantly larger frequency slopes relative to frontal regions, but not consistently relative to posterior sites.

- This pattern indicates that the maximal acceleration of mu frequency is centered over centroparietal areas, rather than being strictly confined to central electrodes, consistent with the topographical results shown in Fig. 3a of the main manuscript.

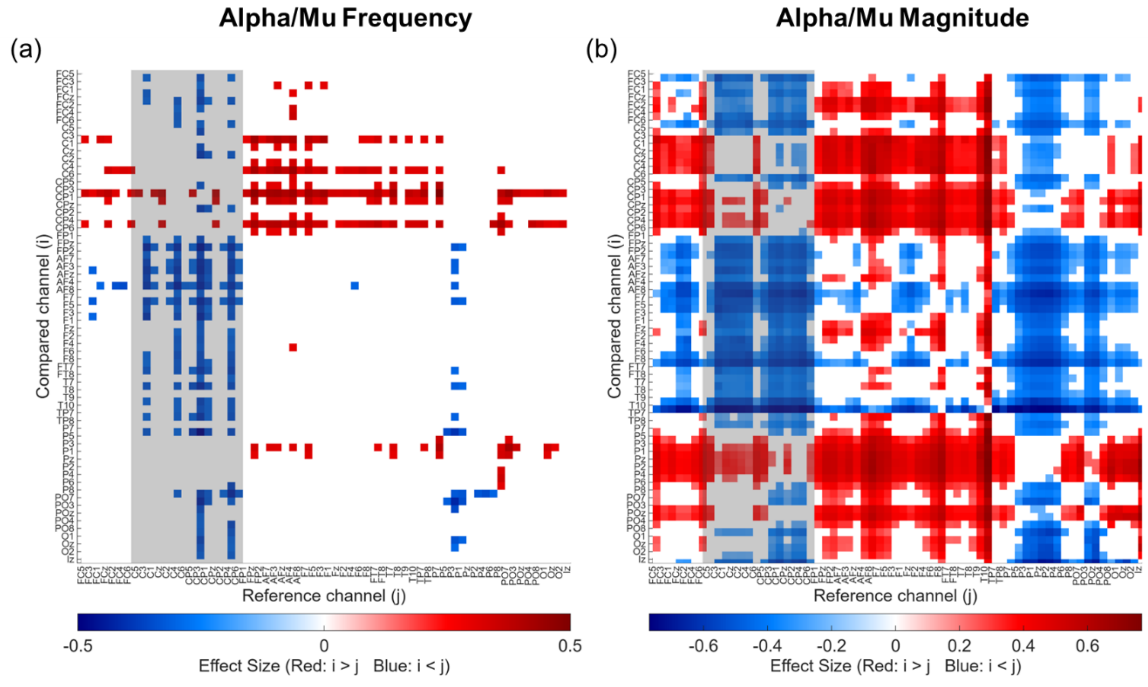

Figure S4. Paired effect-size matrices (Cohen's dz) comparing session-long slopes across electrodes, computed across participants. Each cell represents the paired difference in slope magnitude between a reference channel (j, x-axis) and a compared channel (i, y-axis). Red values indicate that slopes at channel i are larger than at channel j, whereas blue values indicate the opposite. Only statistically significant comparisons after Benjamini–Hochberg FDR correction are shown. The light-grey shaded area denotes electrodes over central and centroparietal regions. **(a)** Frequency slope comparisons. The strongest positive differences are centered over centroparietal electrodes, which exhibit significantly larger frequency increases than both frontal and posterior regions, while central electrodes differ mainly from frontal sites. **(b)** Magnitude slope comparisons. Both central and centroparietal electrodes show significantly larger magnitude increases than frontal regions. Compared to posterior regions, centroparietal sites exhibit consistently larger magnitude slopes, whereas central electrodes show weaker or channel-specific differences.

Magnitude slope comparisons (Fig. S4b):

- Compared to frontal regions, both central and centroparietal electrodes exhibit significantly larger magnitude slopes, indicating stronger session-long increases in alpha/mu amplitude over sensorimotor-related regions (as seen in Fig. 3b of the main manuscript).

- When compared to posterior regions, centroparietal sites show consistently larger magnitude slopes.
- In contrast, central electrodes exhibit only limited or channel-specific differences relative to posterior areas, with no uniform central–posterior separation.

Effect of bandpass selection on alpha/mu frequency and magnitude slope topographies

When the selected frequency band is not well aligned with a participant's dominant alpha/mu peak, the estimator may bias the tracked instantaneous frequency toward the spectral centroid within the analysis band rather than the true resonant frequency of the underlying oscillatory process. This mismatch can introduce systematic biases in the estimated frequency and magnitude slopes. Because all electrodes within a given subject share similar underlying spectral properties, such as dominant alpha/mu frequency and bandwidth, any misalignment between the selected analysis band and the subject's oscillatory activity introduces a subject-level bias that affects all channels in a similar manner. As a result, these biases manifest as subject-specific global offsets in slope estimates that are visible across all electrodes. Importantly, the spatial structure of the slopes is often still present, but it becomes superimposed on a global offset that can dominate group averages and obscure spatial contrasts unless subject-wise normalization (e.g., mean removal) is applied. While subject-wise mean removal can be useful for isolating spatial contrasts when subject-specific offsets dominate, it also removes information about global frequency or magnitude changes, as is the case for alpha/mu magnitude, which exhibits broad session-long increases.

Such an example is shown in Fig. S5 for the Schalk2004 dataset, where different bandpass selections produce uniformly positive (e.g., [7–12] Hz) or uniformly negative (e.g., [8–13] Hz) alpha/mu frequency slopes across the scalp. Despite this global shift in slope polarity, the relative spatial organization, characterized by stronger central and centroparietal effects relative to frontal and posterior regions, remains qualitatively preserved. This demonstrates that misalignment between the selected band and the dominant oscillatory peak primarily introduces a global, subject-dependent offset in slope estimates rather than altering the underlying spatial pattern. Mean

removal suppresses this offset and reveals the spatial structure, but at the cost of discarding information about global frequency or magnitude changes. Note that the observed session-long increase in alpha/mu magnitude cannot be attributed to subject-specific offset artifacts arising from band selection. Unlike frequency slopes, magnitude increases were spatially and spectrally consistent across datasets and participants, and remained positive across a wide range of bandpass choices. Similar patterns were obtained using the Hilbert Transform (Fig. S6). To further verify that these magnitude effects reflect genuine physiological changes rather than estimator bias or baseline offsets, we performed an independent validation using conventional spectral analysis. Specifically, we compared power spectral density estimates obtained via Welch’s method between the first and last 5 minutes of each recording. For each participant and channel, we extracted the alpha peak frequency and corresponding alpha power from the power spectral density (PSD) and directly compared early versus late segments. This analysis consistently revealed increased alpha power toward the end of the session, confirming that the magnitude increases observed in the EKF-based analysis reflect real session-long changes in oscillatory strength rather than artifacts of band misalignment or subject-specific baseline shifts (Fig. S7). We note that the values obtained from this analysis reflect difference values over the first and last 5-minute windows and thus may be underestimated.

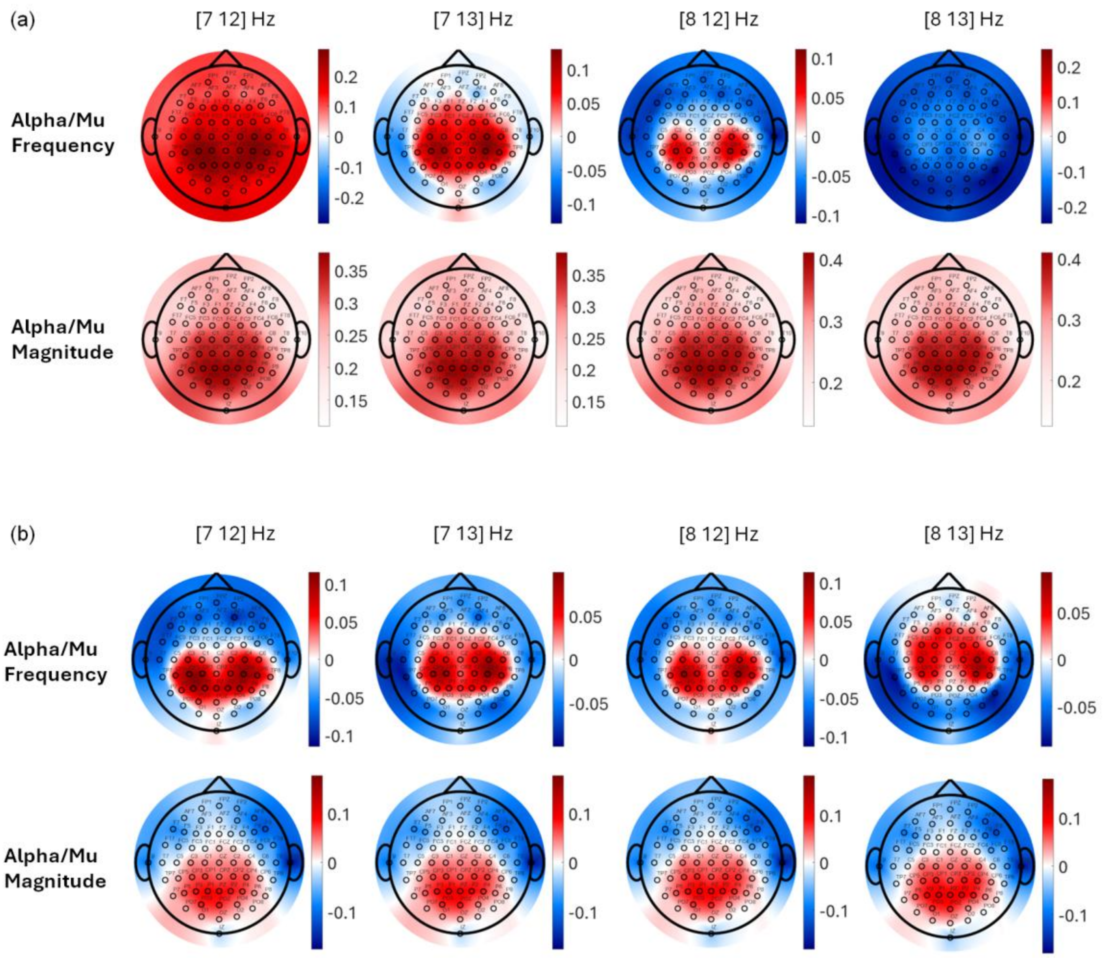

Figure S5. Topographical maps show slopes of EKF-estimated alpha/mu frequency (top rows) and magnitude (bottom rows) across four candidate bandpass ranges ([7–12], [7–13], [8–12], and [8–13] Hz). Panel **(a)** shows slopes computed from raw (non-normalized) trajectories, whereas panel **(b)** shows slopes after subject-wise mean removal to isolate spatial contrasts. Red colors indicate increases over the session, and blue colors indicate decreases. The figure illustrates that bandpass selection strongly affects the global offset of estimated slopes: bands slightly below the dominant alpha/mu peak yield globally positive frequency slopes, while bands above the peak yield globally negative slopes. Importantly, the underlying spatial structure, characterized by central/centroparietal frequency increases and posterior/frontal slowing, remains preserved across bands and becomes more apparent after mean removal. In contrast, alpha/mu magnitude exhibits robust, spatially consistent increases across all tested bands, indicating broad session-long amplitude growth that is less sensitive to bandpass choice. Panel (b) however, depicts the spatial pattern after this global growth has been removed.

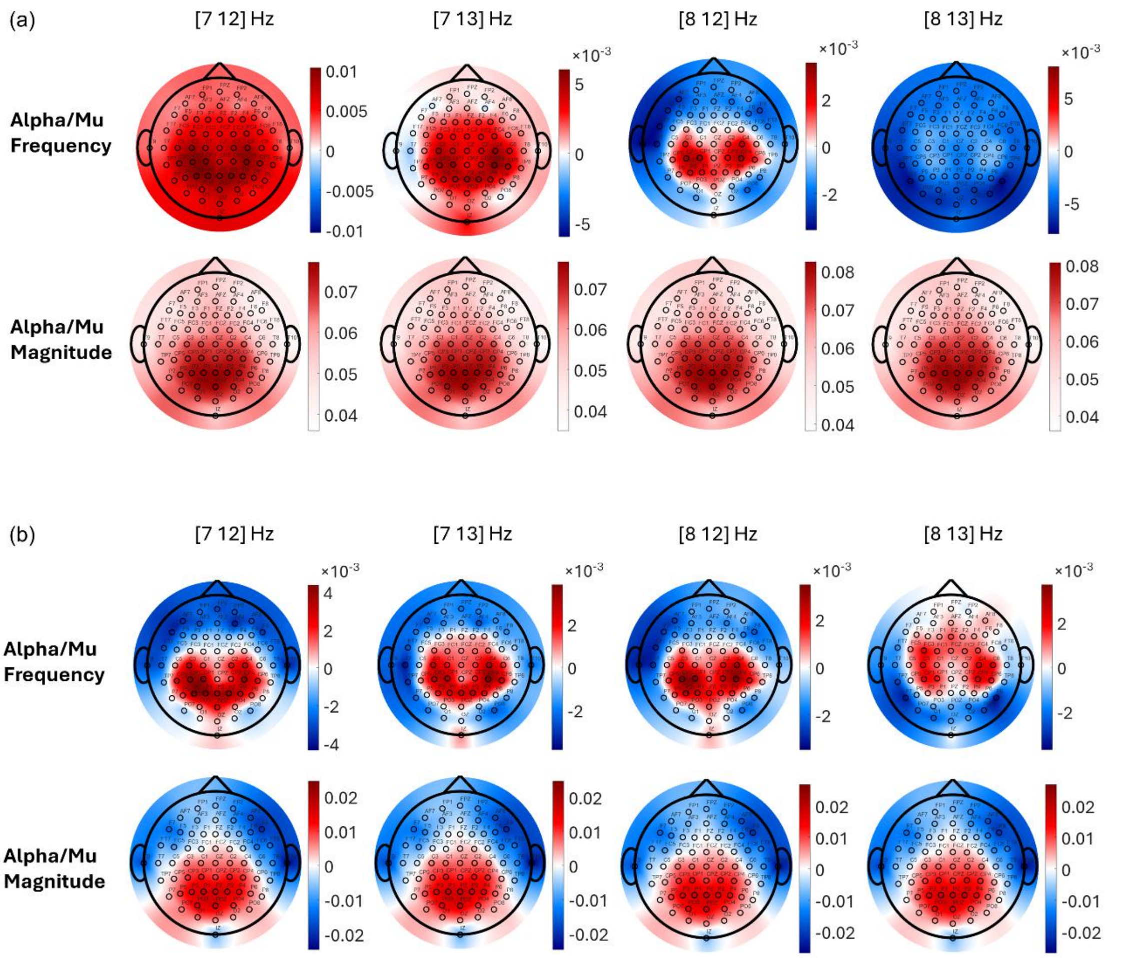

Figure S6. Topographical maps show slopes of HT-estimated alpha/mu frequency (top rows) and magnitude (bottom rows) across four candidate bandpass ranges ([7–12], [7–13], [8–12], and [8–13] Hz). Panel **(a)** shows slopes computed from raw (non-normalized) trajectories, whereas panel **(b)** shows slopes after subject-wise mean removal to isolate spatial contrasts. Red colors indicate increases over the session, and blue colors indicate decreases. The figure illustrates that bandpass selection strongly affects the global offset of estimated slopes: bands slightly below the dominant alpha/mu peak yield globally positive frequency slopes, while bands above the peak yield globally negative slopes. Importantly, the underlying spatial structure, characterized by central/centroparietal frequency increases and posterior/frontal slowing, remains preserved across bands and becomes more apparent after mean removal. In contrast, alpha/mu magnitude exhibits robust, spatially consistent increases across all tested bands, indicating broad session-long amplitude growth that is less sensitive to bandpass choice. Panel (b) however, depicts the spatial pattern after this global growth has been removed.

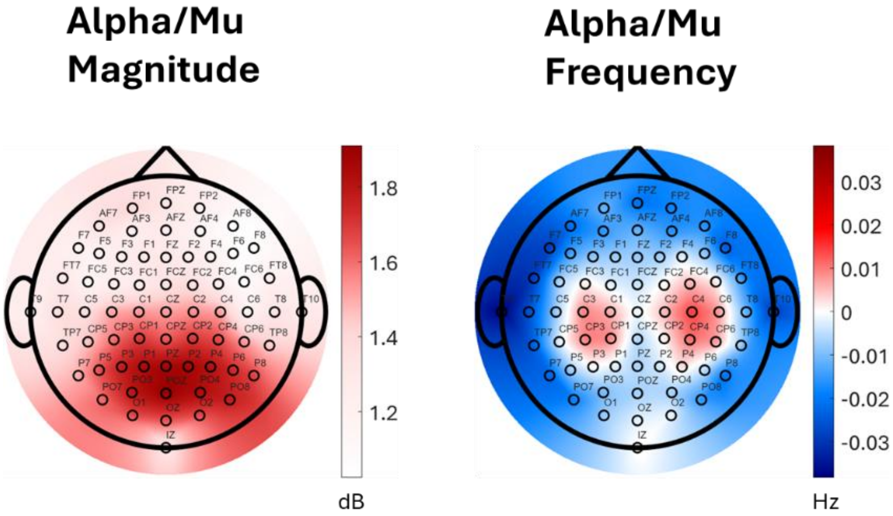

Figure S7. PSD-based validation of alpha/mu magnitude and frequency changes (Schalk2004 dataset). Topographical maps show differences between the last 5 minutes and the first 5 minutes (last – first) of the recording, computed using Welch's power spectral density estimation. Left: change in alpha/mu-band power, obtained by extracting peak alpha/mu power within the dataset-specific band (i.e., [8 12] Hz for Schalk2004) for each electrode. Right: change in alpha/mu resonant frequency, extracted from the PSD within the same band.

SI References

1. Rao BD. Adaptive IIR filtering using cascade structures. Proceedings of 27th Asilomar Conference on Signals, Systems and Computers. 1993. pp. 194–198.
2. Nehorai A, Starer D. Adaptive pole estimation. IEEE Trans Acoust. 1990;38: 825–838.
3. Jackson L, Wood S. Linear prediction in cascade form. IEEE Trans Acoust. 1978;26: 518–528.
4. Ljung L. System identification: theory for the user. Englewood Cliffs. 1987. doi:10.1109/MRA.2012.2192817
5. Nateghi M, Rahbar Alam M, Amiri H, Nasiri S, Sameni R. Model-Based Electroencephalogram Instantaneous Frequency Tracking: Application in Automated Sleep–Wake Stage Classification. Sensors. 2024;24: 7881.
6. Nguyen DP, Wilson MA, Brown EN, Barbieri R. Measuring instantaneous frequency of local field potential oscillations using the Kalman smoother. J Neurosci Methods. 2009;184: 365–374.
7. Simon D. Optimal state estimation: Kalman, H infinity, and nonlinear approaches. John Wiley & Sons; 2006.
8. Golberg DE. Genetic algorithms in search, optimization, and machine learning. Addison Wesley. 1989;1989: 36.
9. Kostoglou K, Müller-Putz GR. Motor-Related EEG Analysis Using a Pole Tracking Approach. IEEE Transactions on Neural Systems and Rehabilitation Engineering. 2024. doi:10.1109/TNSRE.2024.3483294
10. Kostoglou K, Lunglmayr M. Root tracking using time-varying autoregressive moving average models and sigma-point Kalman filters. EURASIP J Adv Signal Process. 2020;2020: 6. doi:10.1186/s13634-020-00666-7
11. Benjamini Y, Hochberg Y. Controlling the false discovery rate: a practical and powerful approach to multiple testing. Journal of the Royal statistical society: series B (Methodological). 1995;57: 289–300.
12. Oppenheim A V. Discrete-time signal processing. Pearson Education India; 1999.
