# Supplementary figures and images for: Opposing cortical forces: Alpha slowing and sensorimotor mu acceleration during motor-related BCI training
Source: PLoS Comput Biol. 2026 Apr 1;22(4):e1014112. doi: 10.1371/journal.pcbi.1014112 (PMC13065009; doi:10.1371/journal.pcbi.1014112)

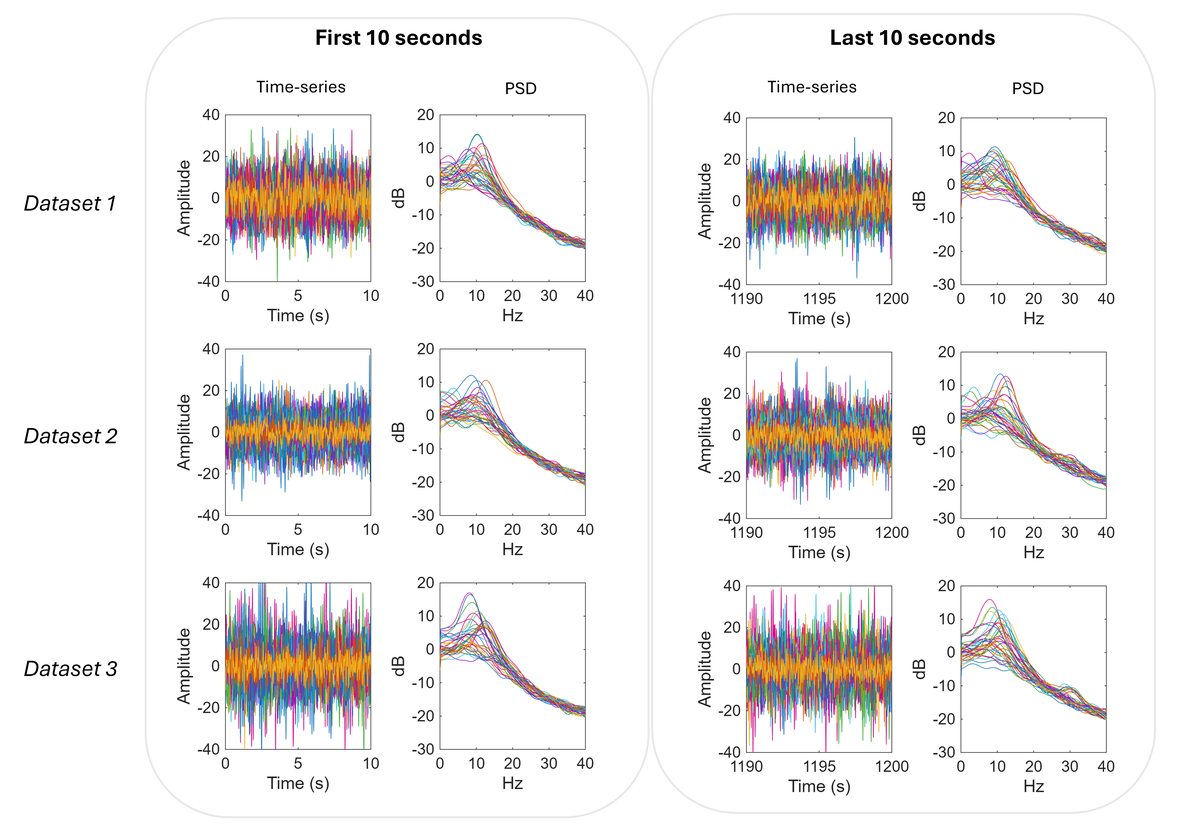

Supplement: S1 Fig — For each dataset (rows), the first ten (left column) and the last ten seconds (right column) of the multichannel signal are shown. Time-series plots display all channels overlaid, illustrating the broadband, noise-embedded oscillatory activity. Corresponding PSDs were estimated using Welch’s method (0.5-s Hanning window, 85% overlap) and show the combined theta, alpha, and beta components embedded in a 1/f-like background. Differences between the first and last segments reflect the imposed session-long changes in oscillatory frequency and magnitude. (TIF) [file pcbi.1014112.s001.tif]

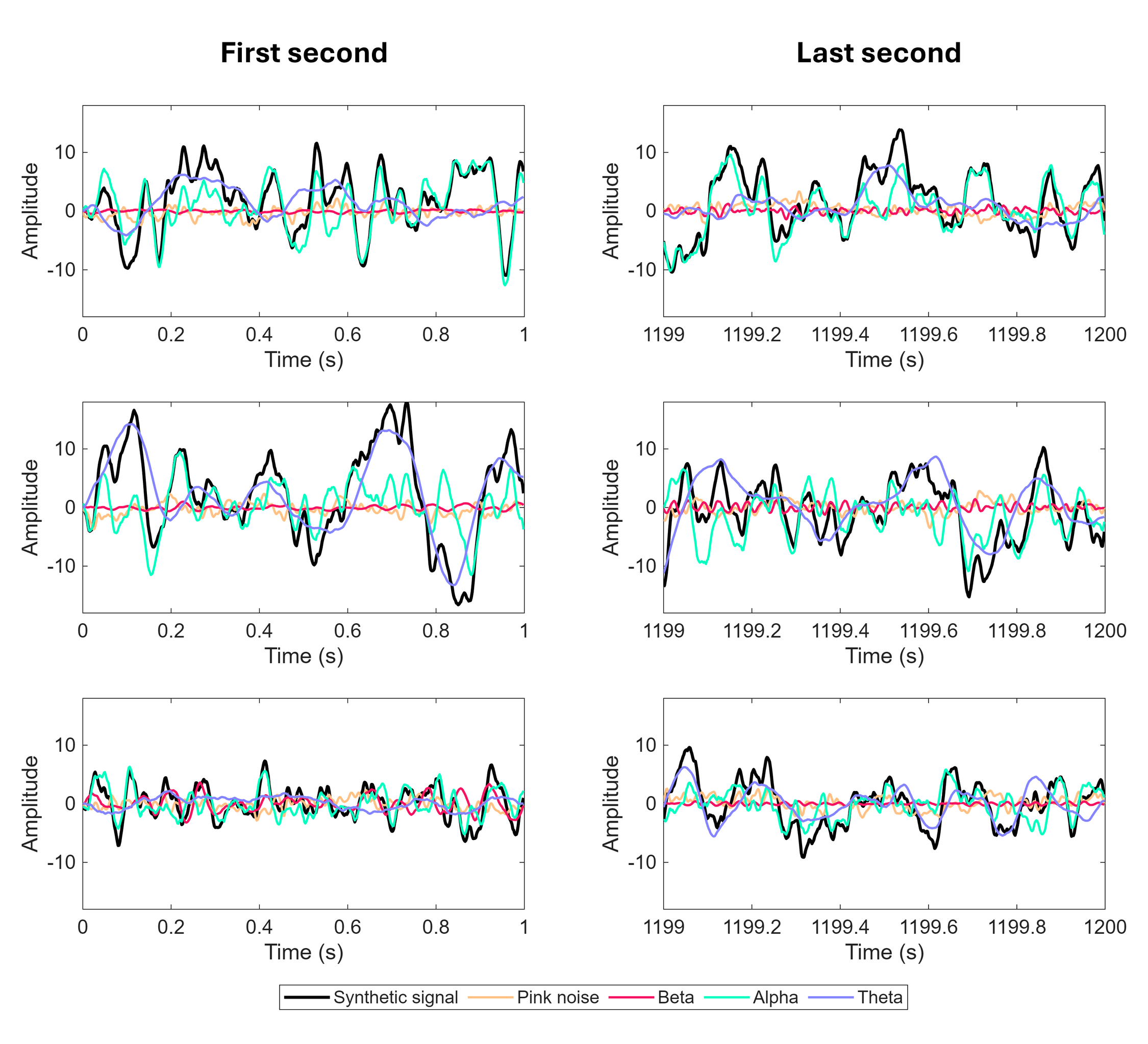

Supplement: S2 Fig — For each channel, the left column shows the first second of the simulations, and the right column shows the last second. The black trace denotes the final synthetic EEG signal, constructed as a weighted sum of band-limited components: pink noise (orange), theta (purple), alpha/mu (teal), and beta (red) (as in Eq. (11) of the Supporting Information). (TIF) [file pcbi.1014112.s002.tif]

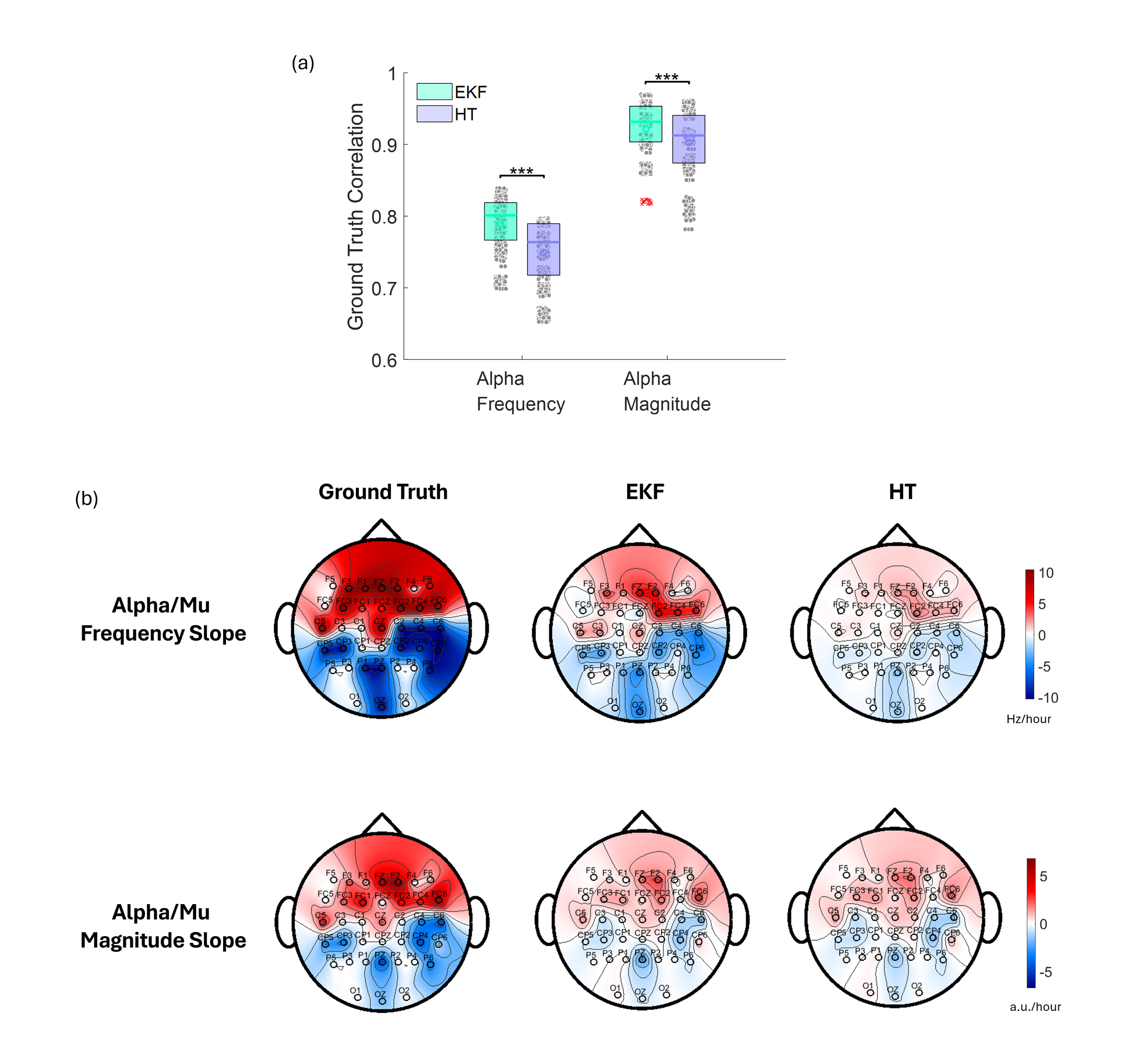

Supplement: S3 Fig — Each boxplot summarizes results across all tested preprocessing configurations, including variations in bandpass filter order, cutoff frequencies (fₘᵢₙ and fₘₐₓ), resampling rates, and the presence or absence of channel-wise standardization. Statistically significant differences are denoted with asterisks (***p < 0.001). (b) EEG topographical maps of alpha/mu-band frequency and magnitude slopes, visualizing spatial trends in ground truth, EKF-based estimates, and HT estimates from one representative synthetic EEG dataset. Red hues indicate increasing slopes (i.e., rising frequency or magnitude over time), while blue hues indicate decreasing slopes. The top row displays alpha/mu frequency slope (Hz/hour) distributions, and the bottom row displays magnitude slope (a.u./hour) distributions. Data were bandpass filtered between [6 14] Hz using a 5th-order Butterworth filter at 120 Hz sampling rate, with no standardization. The EKF method more accurately reproduced ground truth patterns than HT, with frequency slope correlations of 0.826 (EKF) vs. 0.792 (HT), and magnitude slope correlations of 0.935 (EKF) vs. 0.914 (HT). (TIF) [file pcbi.1014112.s003.tif]

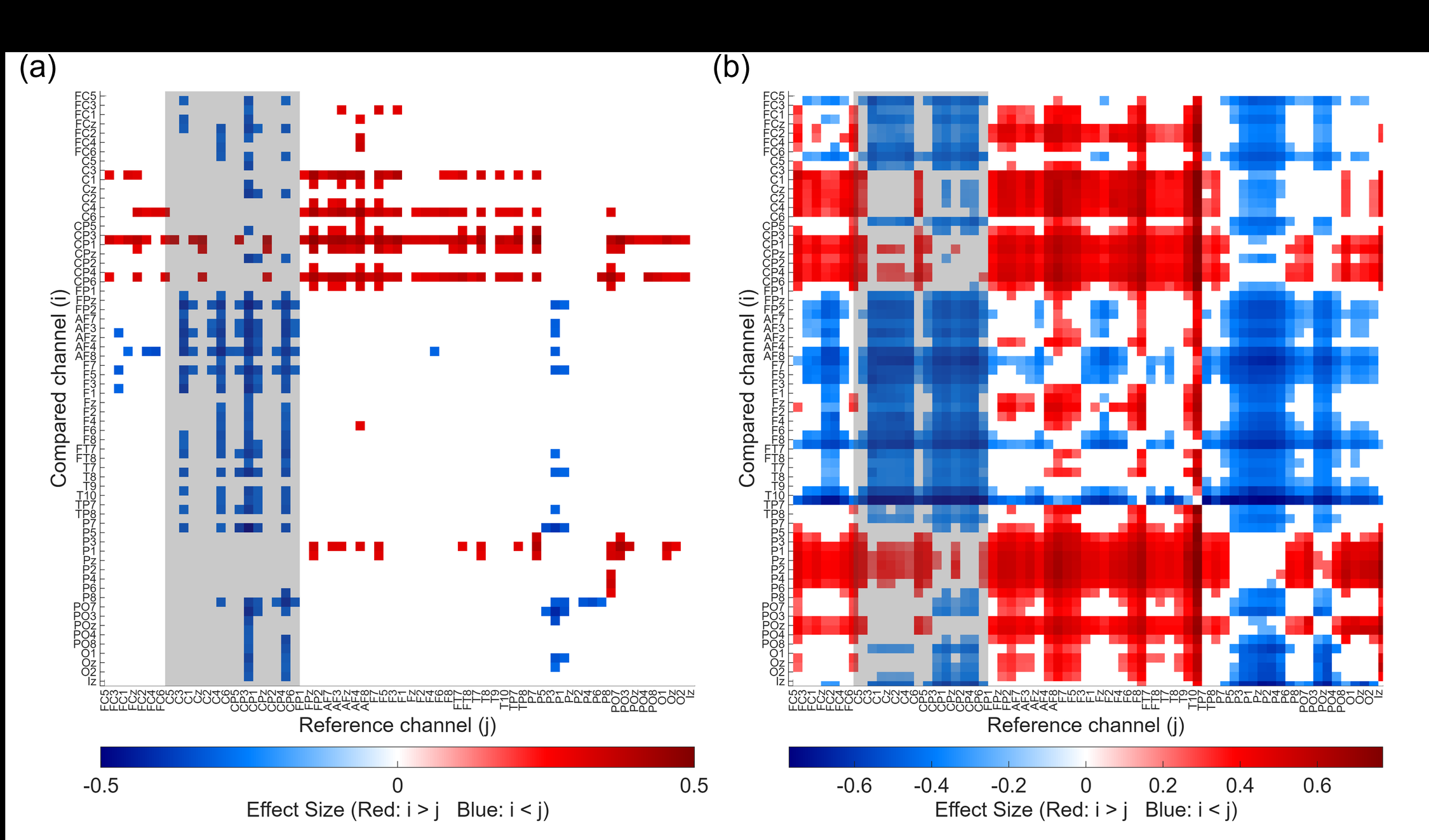

Supplement: S4 Fig — Each cell represents the paired difference in slope magnitude between a reference channel (j, x-axis) and a compared channel (i, y-axis). Red values indicate that slopes at channel i are larger than at channel j, whereas blue values indicate the opposite. Only statistically significant comparisons after Benjamini–Hochberg FDR correction are shown. The light-grey shaded area denotes electrodes over central and centroparietal regions. (a) Frequency slope comparisons. The strongest positive differences are centered over centroparietal electrodes, which exhibit significantly larger frequency increases than both frontal and posterior regions, while central electrodes differ mainly from frontal sites. (b) Magnitude slope comparisons. Both central and centroparietal electrodes show significantly larger magnitude increases than frontal regions. Compared to posterior regions, centroparietal sites exhibit consistently larger magnitude slopes, whereas central electrodes show weaker or channel-specific differences. (TIF) [file pcbi.1014112.s004.tif]

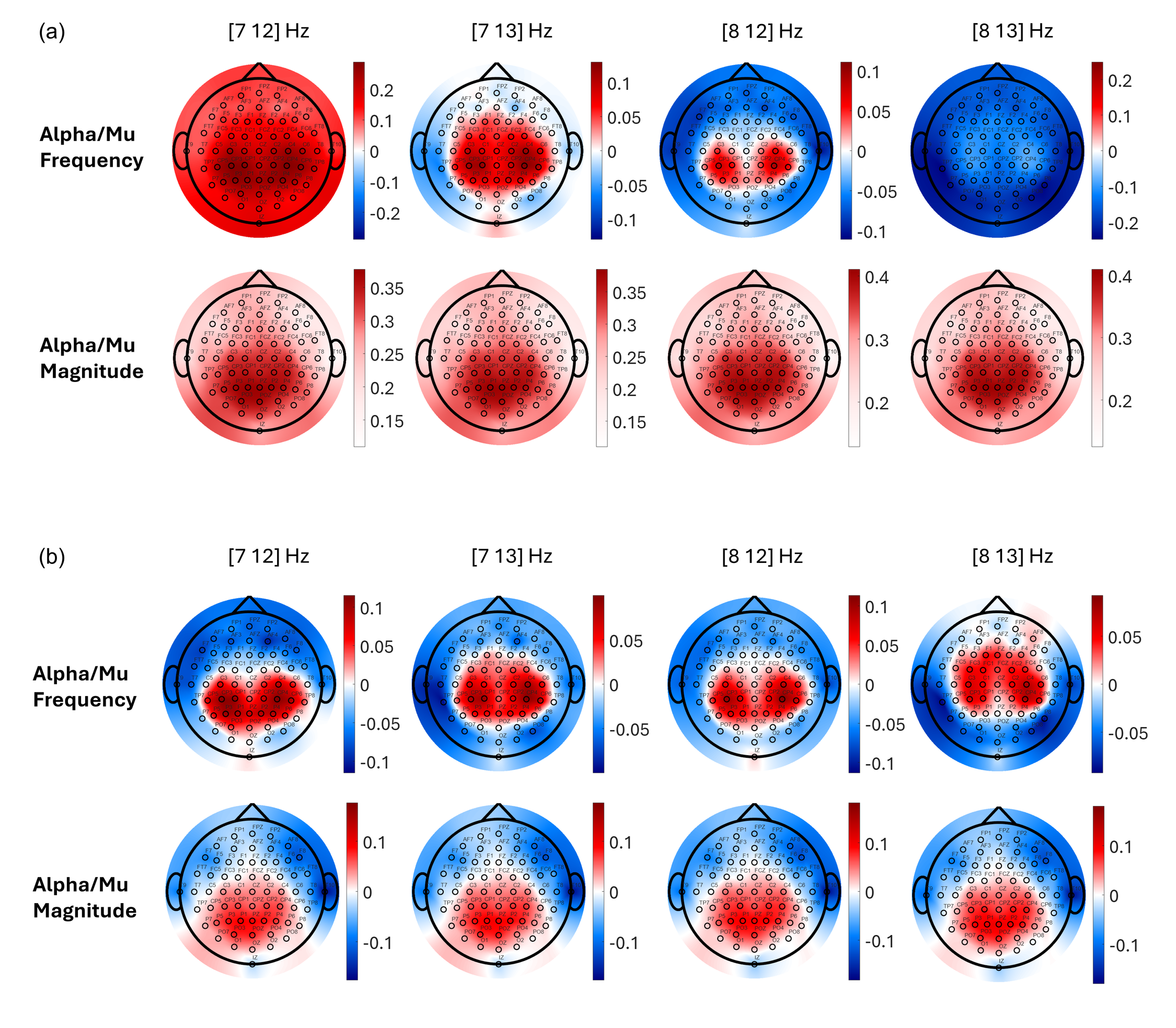

Supplement: S5 Fig — Panel (a) shows slopes computed from raw (non-normalized) trajectories, whereas panel (b) shows slopes after subject-wise mean removal to isolate spatial contrasts. Red colors indicate increases over the session, and blue colors indicate decreases. The Fig illustrates that bandpass selection strongly affects the global offset of estimated slopes: bands slightly below the dominant alpha/mu peak yield globally positive frequency slopes, while bands above the peak yield globally negative slopes. Importantly, the underlying spatial structure, characterized by central/centroparietal frequency increases and posterior/frontal slowing, remains preserved across bands and becomes more apparent after mean removal. In contrast, alpha/mu magnitude exhibits robust, spatially consistent increases across all tested bands, indicating broad session-long amplitude growth that is less sensitive to bandpass choice. Panel (b) however, depicts the spatial pattern after this global growth has been removed. (TIF) [file pcbi.1014112.s005.tif]

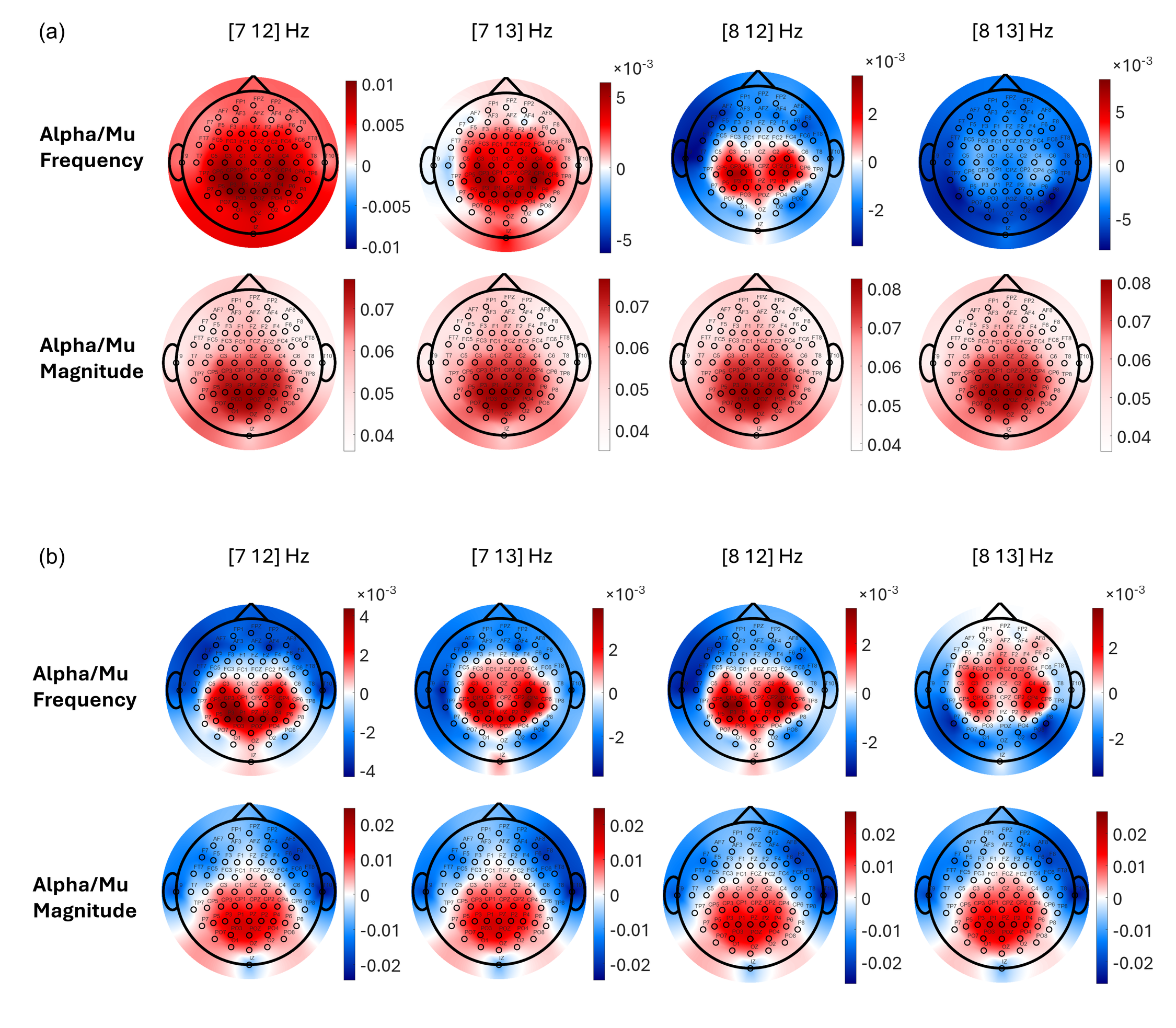

Supplement: S6 Fig — Panel (a) shows slopes computed from raw (non-normalized) trajectories, whereas panel (b) shows slopes after subject-wise mean removal to isolate spatial contrasts. Red colors indicate increases over the session, and blue colors indicate decreases. The Fig illustrates that bandpass selection strongly affects the global offset of estimated slopes: bands slightly below the dominant alpha/mu peak yield globally positive frequency slopes, while bands above the peak yield globally negative slopes. Importantly, the underlying spatial structure, characterized by central/centroparietal frequency increases and posterior/frontal slowing, remains preserved across bands and becomes more apparent after mean removal. In contrast, alpha/mu magnitude exhibits robust, spatially consistent increases across all tested bands, indicating broad session-long amplitude growth that is less sensitive to bandpass choice. Panel (b) however, depicts the spatial pattern after this global growth has been removed. (TIF) [file pcbi.1014112.s006.tif]

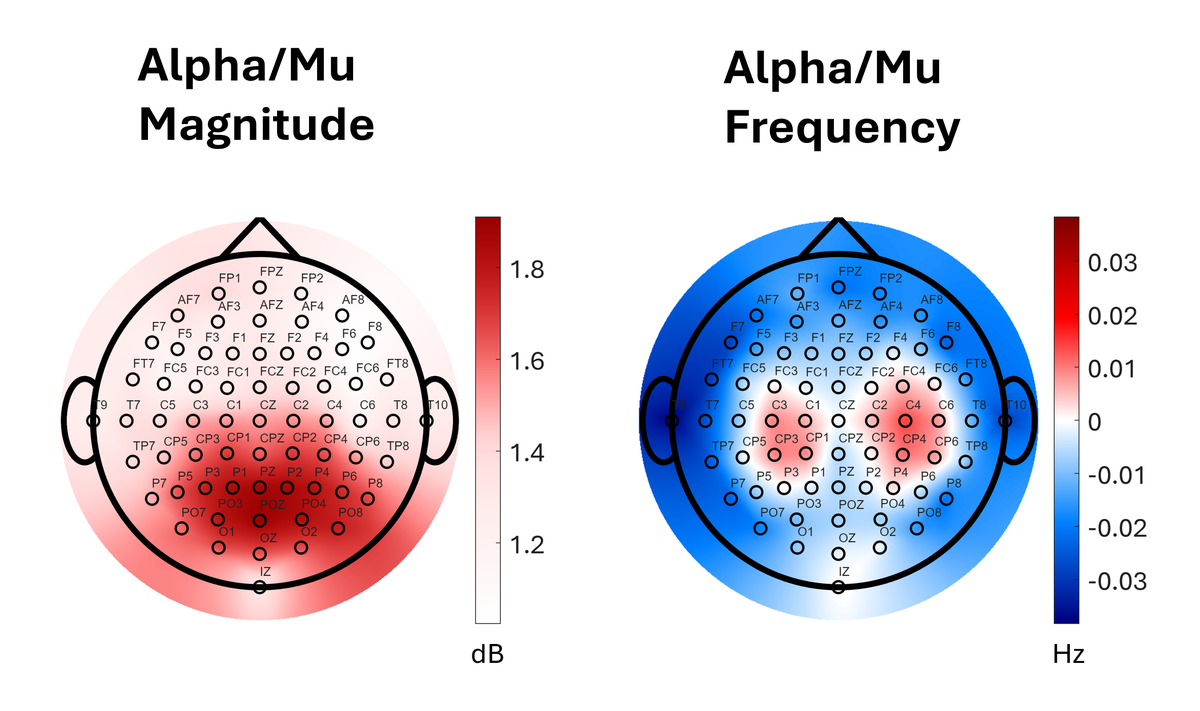

Supplement: S7 Fig — Topographical maps show differences between the last 5 minutes and the first 5 minutes (last – first) of the recording, computed using Welch’s power spectral density estimation. Left: change in alpha/mu-band power, obtained by extracting peak alpha/mu power within the dataset-specific band (i.e., [8 12] Hz for Schalk2004) for each electrode. Right: change in alpha/mu peak frequency, extracted from the PSD within the same band. (TIF) [file pcbi.1014112.s007.tif]
